# Apium extract alleviates indomethacin-induced gastric ulcers in rats via modulating the VEGF and IK-κB/NF-κB p65 signaling pathway: insights from in silico and in vivo investigations

**DOI:** 10.1186/s12906-023-04333-w

**Published:** 2024-02-14

**Authors:** Dalia H. Abu-Baih, Alshymaa Abdel-Rahman Gomaa, Nada Mohamed Abdel-Wahab, Enas Reda Abdelaleem, Azza M. Abdel Zaher, Noha F. Hassan, Gerhard Bringmann, Usama Ramadan Abdelmohsen, Faisal H. Altemani, Naseh A. Algehainy, Fatma Alzahraa Mokhtar, Miada F. Abdelwahab

**Affiliations:** 1 Department of Biochemistry, Faculty of Pharmacy, Deraya University, New Minia, 61111 Egypt; 2 Department of Pharmacognosy, Faculty of Pharmacy, Minia University, Minia, 61519 Egypt; 3 Department of Pathology, Faculty of Medicine, Minia University, Minia, Egypt; 4 Department of Pharmacology and Toxicology, Faculty of Pharmacy, Modern University for Technology and Information, Cairo, 11571 Egypt; 5 Institute of Organic Chemistry, University of Würzburg, Am Hubland, Würzburg, 97074 Germany; 6 Department of Pharmacognosy, Faculty of Pharmacy, Deraya University, Minia, 61111 Egypt; 7 Department of Medical Laboratory Technology, Faculty of Applied Medical Sciences, University of Tabuk, Tabuk, 71491 Saudi Arabia; 8 Fujairah Research Centre, Sakamkam Road, Fujairah, United Arab Emirates; 9 Department of pharmacognosy, Faculty of pharmacy, El Saleheya El Gadida University, El Saleheya El Gadida, 44813 Sharkia Egypt

**Keywords:** *Apium graveolens* L., Gastric ulcer, Metabolomic profiling, Network pharmacology, Inflammatory cytokines

## Abstract

**Background:**

Gastric ulcers represent a worldwide health problem, characterized by erosions that affect the mucous membrane of the stomach and may even reach the muscular layer, leading to serious complications. Numerous natural products have been assessed as anti-ulcerogenic agents, and have been considered as new approaches for treatment or prevention of gastric ulcers. The present research investigated the preventive benefits of *Apium graveolens* L. (Apiaceae), known as celery, seed extract towards indomethacin-induced ulceration of the stomach in rats.

**Methods:**

Metabolomic profiling, employing liquid chromatography coupled to high-resolution electrospray ionization mass spectrometry (LC-HR-ESI–MS), was implemented with the aim of investigating the chemical profile of the seeds. Histopathological analysis of gastric tissues, as well as assessment of numerous inflammatory cytokines and oxidative stress indicators, confirmed the in vivo evaluation.

**Results:**

The prior treatment with *A. graveolens* seed extract resulted in a substantial reduction in the ulcer index when compared to the indomethacin group, indicating an improvement in stomach mucosal injury. Moreover, the gastroprotective effect was demonstrated through examination of the oxidative stress biomarkers which was significantly attenuated upon pre-treatment with *A. graveolens* seed extract. Vascular endothelial growth factor (VEGF), a fundamental angiogenic factor that stimulates angiogenesis, was markedly inhibited by indomethacin. *A. graveolens* seed extract restored this diminished level of VEGF. The dramatic reductions in NF-κB protein levels indicate a considerable attenuation of the indomethacin-induced IKκB/NF-κB p65 signaling cascade. These activities were also correlated to the tentatively featured secondary metabolites including, phenolic acids, coumarins and flavonoids, previously evidenced to exert potent anti-inflammatory and antioxidant activities. According to our network pharmacology study, the identified metabolites annotated 379 unique genes, among which only 17 genes were related to gastric ulcer. The PTGS2, MMP2 and PTGS1 were the top annotated genes related to gastric ulcer. The top biological pathway was the VEGF signaling pathway.

**Conclusion:**

*A. graveolens* seed extract possesses significant anti-ulcer activity, similar to famotidine, against gastric lesions induced by indomethacin in rats. It is worth highlighting that the extract overcomes the negative effects of conventional chemical anti-secretory drugs because it does not lower stomach acidity.

**Supplementary Information:**

The online version contains supplementary material available at 10.1186/s12906-023-04333-w.

## Introduction

The histological definition of gastric ulcerative disease is mucous membrane injury that affects of the stomach superficial or deeper muscularis mucosa [1, 2]. The epidemiological data has unveiled remarkable rates of incidence and prevalence across diverse geographical regions. It has been demonstrated that the worldwide occurrence of gastric ulcer amounted to approximately 8.1 million individuals, signifying a notable surge of 25.8% compared to the figures recorded in 1990 [3]. The disease origin is complex and multifaceted, largely due to an imbalance between endogenous protective and aggressive factors to the stomach mucosa [4]. Defense factors include mucus, cytoprotective prostaglandins, nitric oxide, bicarbonate, the endogenous antioxidant system, and a sufficient blood flow [5]. The most important aggressive agents involve hydrochloric acid, pepsin, bile reflux, reactive oxygen species (ROS), reduced blood flow, and infection with *Helicobacter pylori* [6]. Additionally, exogenous aggressive factors including stress, alcoholism, smoking, nutritional deficiencies and the frequent and indiscriminate usage of non-steroidal anti-inflammatory drugs (NSAIDs) contribute to a large extent to the development of ulceration [7].

Because of their acidic nature, NSAIDs cause primary mucosal irritation and secondary or indirect harm to the gastric mucosa by inhibiting the synthesis of gastro-protective thromboxane and prostaglandins [8]. Aside from that, all of the previously mentioned noxious agents, including NSAIDs, might contribute to ulcer formation via producing various kinds of ROS, which increase the release of several inflammatory cytokines, including nuclear factor kappa (NF-κB) and tumor necrosis factor-α (TNF-α) [9].

Gastric ulcer complications can cause gastroduodenal perforation, bleeding, and obstruction if they are not addressed or treated appropriately [10]. Treatment often consists of anti-secretory medicines such as H2-receptor blockers and proton pump inhibitors, as well as antibiotics such as clarithromycin and/or metronidazole if an *H. pylori* infection is demonstrated to be active by laboratory testing [11]. Nonetheless, because of the bad responses and limited efficacy of currently available medications, managing stomach ulcers is one of the most difficult challenges, with a large economic impact on public health systems [12]. Proton pump inhibitors have been linked to major side effects such as fractures, renal disease, greater vulnerability to certain infections, and calcium, magnesium, and vitamin B12 deficiency [13]. Many nations have phased out the H2-blocker ranitidine due to the identification of above-the-limit amounts of the carcinogenic pollutant N-nitroso-dimethylamine (NDMA) [14]. Furthermore, there is evidence that proton pump inhibitors can enhance the intestinal injury produced by NSAIDs by changing the microbial composition of the intestine [15] and increase the risk of gastric cancer [16]. Thus, traditional medicine may be of significant interest in the search for non-toxic, easily accessible, and economical anti-ulcer drug.

Various herbal medicines are prevalently employed to treat and/or prevent many diseases including gastric ulcers [17–21]. The anti-ulcerogenic and gastroprotective potentials of natural products are attributed mostly to their diverse metabolic profile and antioxidant properties [22–24]. Therefore, complementary treatments could be developed to mitigate the ulcerative diseases and inhibit recurrences with less side effects, higher efficacy, and affordability compared to synthetic drugs [25].

*Apium graveolens* L. (Apiaceae, Umbelliferae), commonly known as celery, is an aromatic biennial herb indigenous to Southern Europe, Asia and Africa. It has long been consumed as medicine or food in the form of entire herb, leaves, stalks, seeds or seed [26, 27]. The aerial parts extract as well as the essential oil have been demonstrated to exhibit a significant antiulcer effect [27, 28]. The findings indicated that *A. graveolens* has the ability to diminish gastric acid secretion and strengthen the defense factors of gastric mucosa. These anti-secretory and cytoprotective effects are probably mediated by its antioxidant properties through reduction of lipid peroxidation and elevation of gastric mucosal non-protein sulfhydryl groups [28]. In addition to its anti-acid and softening effects [27, 29]. Interestingly, a recent study has demonstrated that celery also exhibits bactericidal activity against *H. pylori* [30].

Seeds of *A. graveolens* have been traditionally used as a flavoring agent or in treatment of gut diseases, urinary calculi, flatulence, gripping pain and visceral [31, 32]. Moreover, previous pharmacological studies reported that the seeds revealed hypoglycemic [33], bactericidal [34], antifungal [35], nematocidal, mosquitocidal [36], anti-inflammatory [37] and anti-hypertensive [38] activities. Therefore, the present study was designed to investigate the phytochemical composition of the seeds of *A. graveolens* through metabolomic profiling using liquid chromatography high-resolution electrospray ionization mass spectrometry (LC-HR-ESI–MS), along with in vivo evaluation of the anti-gastric ulcer potential of the seed extract in experimental animals. Furthermore, the pharmacological and biochemical results were substantiated by a histopathological assessment of the gastric tissues, and by an in silico network pharmacology study involving all identified metabolites, in a trial to determine the gene enrichment analysis and to understand the top biological pathway of the anti-gastric ulcer activity.

## Materials and methods

The seeds of *A. graveolens* were purchased from Minia seeds market. Plant materials and experiments were conducted in accordance with relevant institutional, national, and international guidelines.

### Plant material

The seeds of *A. graveolens* were collected in September 2022 from Minia area, Egypt, and were verified by Prof. Dr. Nasser Barakat (Professor of Botany, Faculty of Science, Minia University). A voucher sample (Mn-ph-Cog-040) has been deposited at the herbarium of the Pharmacognosy Department at the Faculty of Pharmacy of Minia University.

### Preparation of extract

Air-dried powdered seeds of *A. graveolens* (500 g) were extracted with methanol. The extract was filtrated and concentrated under reduced pressure at 45°C using a rotavapor to yield 45 g of dry extract.

### Metabolomics analysis

According to the approach described by Hamed et al. [39] and Ahmed et al. [40], liquid chromatography high resolution electrospray ionization mass spectrometry (LC-HR-ESI–MS) was used to perform chemical profiling for *A. graveolens* seed extract. An Acquity Ultra Performance Liquid Chromatography system coupled to a Synapt G2 HDMS quadrupole time-of-flight hybrid mass spectrometer (Waters, Milford, USA) was employed. Ms converter software was used to convert the raw data into positive and negative ionization files. The data mining software MZmine2.20 (Okinawa Institute of Science and Technology Graduate University, Japan) was used to extract and analyze the data. Following the identification of mass ion peaks, the chromatogram builder and deconvolution were performed. The isotopic peaks grouper processed the local minimum search algorithm, and isotopes were identified. The detected constituents were annotated by comparison with the METLIN 2020 and Dictionary of Natural Products 2020 (DNP) databases.

### Investigation of anti-ulcer potential

#### Animals

Adult male Wistar rats (180–200 gm) were procured from the animal house of the Faculty of Pharmacy of Minia University, Minia, Egypt. Acclimatization for the experiment was completed one week prior to the start of the trial, and all circumstances were designed to minimize animal suffering. The experiment was approved by the ethical review board of Faculty of Pharmacy, Minia University in Minia, Egypt, and was carried out in compliance with the Helsinki Declaration criteria [41] and ARRIVE guidelines. The rats were housed in four cages with bedding made of wood shavings. The rats were kept in conventional laboratory settings (22°C, relative humidity 50–55%, and a 12 h light/dark cycle) and had unlimited access to regular rodent food and water. To eliminate changes due to diurnal cycles of potential regulators of stomach functions, all rats were used in the experiment at the same time of day [42].

#### Experimental design

The 24 used rats were divided into four groups (six rats/group).

**Fig. 1:**
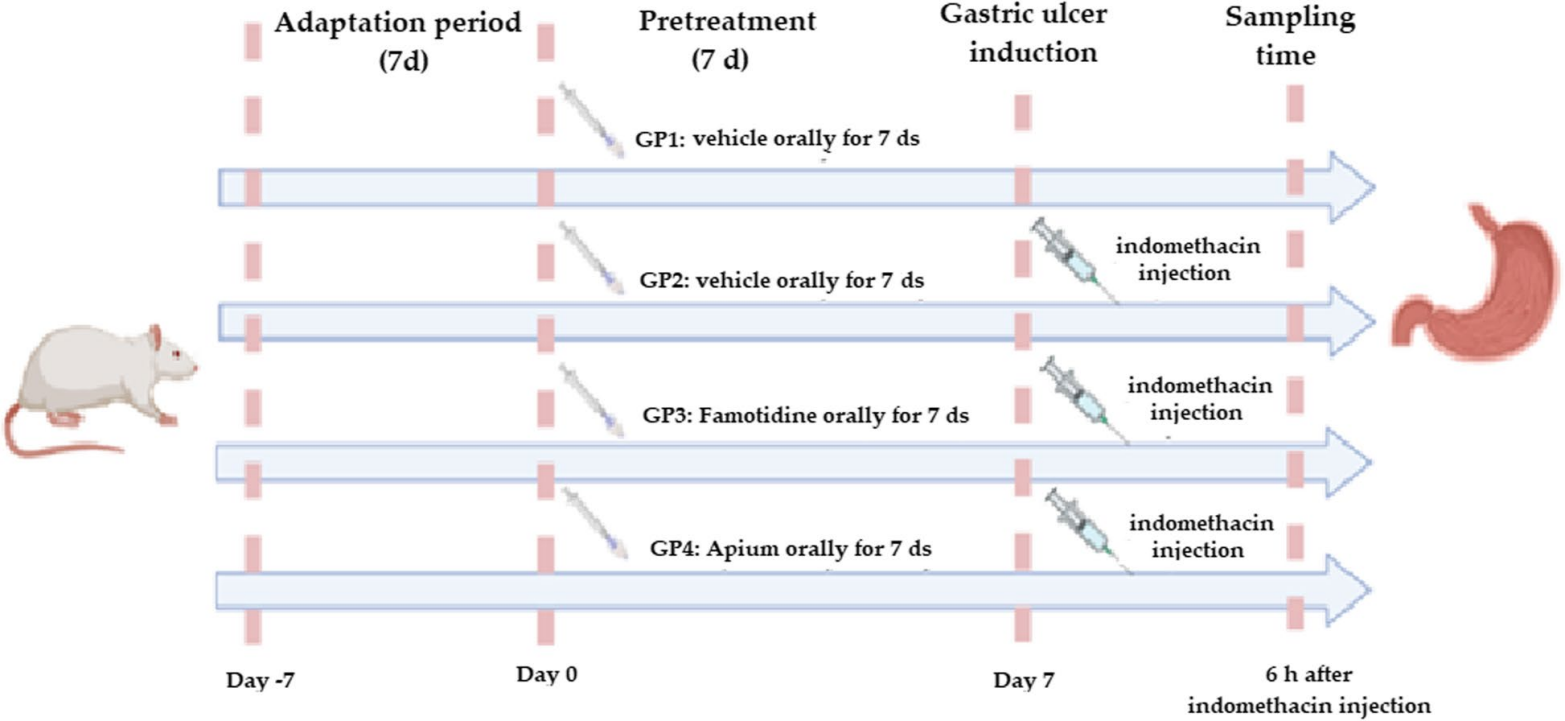
Time course of experimental schedule. Control, ulcer, famotidine treated, and *A. graveolens* treated groups

- Group 1 (the control group) received vehicle (0.5% carboxy methylcellulose (CMC)) (1 mL/rat/once daily, oral gavage) for 7 d.
- Group 2 (the ulcer group) received vehicle (0.5% CMC) (1 mL/rat/once daily, oral gavage) for 7 d, and then indomethacin on day 7.
- Group 3 (the famotidine treated group) received famotidine (10 mg/kg/once per day, oral gavage) suspended in 1 mL of 0.5% CMC for 7 consecutive days followed by indomethacin on day 7.
- Group 4 (the *A. graveolens* treated group) received *A. graveolens* extract (250 mg/kg/once per day, oral gavage) for 7 consecutive days, and then indomethacin on day 7. The experimental design is illustrated in Fig. 1.

Gastric lesions were induced using a version of Djahanguiri [43] approach, with a few alterations. After a 16-h fast (with free access to water), animals were given a single dose of indomethacin (40 mg/kg, oral gavage) [44].

#### Blood and tissue sampling

The animals were anesthetized by thiopental sodium (50 mg/kg), 6 h after indomethacin administration and the stomachs were removed for experimental protocols. The gastric contents were collected by opening the stomachs of the rats along the greater curvature. A segment of the gastric region of every rat was meticulously removed and preserved in a solution of 10% formalin-saline for subsequent histopathological investigations. The remaining portion was rapidly frozen using liquid nitrogen and stored at a temperature of -80℃ for future tissue analyses.

#### Assessment of gross mucosal damage

The gastric cavity of each rat was rinsed with frigid saline solution and gently dried using absorbent filter papers. The stomach, once cleansed, underwent a process of fixation onto a corkboard. Subsequently, digital imaging was done for the stomachs, enabling the assessment of potential harm inflicted upon the mucosal lining. The images underwent analysis using ImageJ software, developed by Wayne Rasband, MD, USA. The software was utilized to measure the areas of ulcerations, and subsequently, the ulceration ratio was determined. The methodology outlined by Szabo and Hollander [45], was followed to calculate the ulcer index (U.I.) for each animal, employing the following formula:

$$\text U.\text I.\left(\%\right)=\frac{Ulcerated \,area}{Total\,stomach \,area}\times 100$$

The percentage of inhibition against ulceration was determined using the formula:

$$Ulcer\,inhibition\,\left(\%\right)=\frac{U.I.\,in\,ulcer\,group-U.I.\,in\,test \,group}{U.I.\,in\,ulcer \,group}\times 100$$

#### Microscopic examination of gastric ulcers

The stomach tissues underwent fixation in a 10% neutral buffered formalin solution for a duration of 1 day. Subsequently, they were subjected to dehydration and embedded in paraffin wax. Following this, the tissues were transversally sectioned using a sledge microtome, resulting in sections with a thickness of 5 µm. Histopathological examination under the light microscope was employed to detect microscopic gastric injury, utilizing hematoxylin and eosin staining (H&E) staining [28].

#### Measurement of gastric acidity

The evaluation of gastric juice pH was conducted, as described by Beiranvand et al. In a concise manner, the gastric content from each rat was subjected to centrifugation at a speed of 5000 revolutions per minute for a duration of 10 min. The resulting supernatant was carefully collected, and subsequently, 1 mL of the supernatant was mixed with an equal volume of distilled water. The pH of this mixture was then determined using a pH meter (Fisherbrand, AB315 benchtop pH meter, Waltham, MA, USA) [46, 47].

#### Assessment of gastric tissue oxidative stress markers

The quantification of malondialdehyde (MDA), a byproduct of lipid peroxidation, in the tissue supernatant was conducted to assess the extent of oxidative stress [48], The measurement was performed using the colorimetric technique developed by Tappel and Zalkin, and the results were reported in units of nanomoles per gram of tissue [49]. The assessment of antioxidant markers involved the quantification of the reduced glutathione (GSH) concentration, the naturally occurring non-enzymatic antioxidant, using the methodology outlined by Ellman. The results were then expressed as millimoles per gram of tissue [50].

#### Gene expression analysis

##### Total RNA extraction

A tissue sample weighing 50 mg was subjected to homogenization using an ultrasonic homogenizer (Sonics-Vibracell, Sonics and Materials Inc., Newtown, Fairfield County, Connecticut, USA). The homogenization was carried out in 0.5 mL of TRIzol reagent (RNA Isolation Reagent, Invitrogen—ThermoFisher Products & Kits, Amresco, LLC-Solon, USA) [44]. Tissue samples were subjected to total RNA isolation following the prescribed protocol provided by the manufacturer. The estimation of RNA yield and purity concentrations was performed [51].

##### Real-time PCR

The Revert Aid H Minus First Strand cDNA Synthesis kit (#K1632, Thermo Scientific Fermentas, St. Leon-Ro, Germany) was utilized in accordance with the provided guidelines from the manufacturer. This kit facilitated the reverse transcription process, wherein an equivalent amount of total RNA was employed in all samples. Maxima SYBR Green qPCR Master Mix (2X) is used for quantitative polymerase chain reaction (qPCR) assays (#K0251, Thermo Scientific Fermentas St. Leon-Ro, Germany). Interleukin-6 (IL-6), Interleukin-1β (IL-1β), TNF-α, and Cyclooxygenase-II (COX-II) play crucial roles in orchestrating the inflammatory cascade and subsequent tissue injury that accompanies the development of gastric ulcers. By quantifying the aforementioned biomarkers within gastric tissues, valuable insights can be gained regarding the underlying inflammatory mechanisms, the extent of inflammation, the efficacy of treatment, and the potential identification of specific targets for therapeutic intervention. Table 1 presents the repertoire of primers employed for qRT-PCR [52]. StepOne™ Real-Time PCR Detection System (Applied Biosystems) [53] was employed to conduct real-time PCR reactions. Intial denaturation step was performed at 95°C for 10 min followed by 40 cycles of 95 °C for 15 s and aneling/extension at 60 °C for 1 min. The quantification of gene expression levels was performed, followed by normalization to the reference gene glyceraldehyde-3-phosphate dehydrogenase (GAPDH), which served as a stable internal control. By employing the comparative Ct method, we assessed the relative abundances of RNA. In order to determine the relative expression, the formula utilized was 2 ^(−ΔΔCt)^ [54].

**Table 1:** Primers used for real-time PCR

Name of gene		Primer sequence
IL-1β	Forward	5`- GTGATGAAAGACGGCACACC-3′
	Reverse	5`- TCCTGGGGAAGGCATTAGGA-3′
GAPDH	Forward	5`-CTCTCTGCTCCTCCCTGTTC-`3
	Reverse	5`-CGACATACTCAGCACCAGCA-`3
IL-6	Forward	5`-TCTGGTCTTCTGGAGTTCCGT-3′
	Reverse	5`-GGATGGTCTTGGTCCTTAGCC-3′
TNF-α	Forward	5`-CCTCTCTGCCATCAAGAGCC-3′
	Reverse	5`- GGCTGGGTAGAGAACGGATG-3′
COX-2	Forward	5` TTCGGGAGCACAACAGAGTG 3′
	Reverse	5` CAGCGGATGCCAGTGATAGA 3′
iNOS	Forward	5′- CACCACCCTCCTTGTTCAAC -3′
	Reverse	5′- CAATCCACAACTCGCTCCAA -3′

#### Determination of gastric tissue protein expression of VEGF and NF-κB using ELISA

Gastric VEGF level was estimated using a commercially available ELISA kit (catalog # ab100787, Abcam, MA, USA). Additionally, gastric tissue was lysed and phospho-NFκB p65(Ser536)/total NF-κB p65 content, a critical regulator of immune and inflammatory responses particularly implicated in gastric ulcer pathogenesis, was estimated using an ELISA kit (catalog # PEL-NFKBP65-S536-T) obtained from Ray Biotech (GA, USA) [55].

#### Statistical analysis

The findings are presented as mean value plus or minus the standard error of mean (SEM). Multiple comparisons were conducted employing a one-way analysis of variance (ANOVA) followed by Tukey's test for multiple comparisons. The statistical analyses were performed using GraphPad Prism 8 software (GraphPad Software Inc., La Jolla, CA, USA). When the probability *p* values ≤ 0.05, differences were considered significant.

### Network pharmacology and gene ontology analysis

Network Pharmacology study was used to construct the relationship between plant metabolites, annotated genes, and gastric ulcer disease and determine the gene enrichment analysis using different databases; PubChem, Binding DB, DisGeNET, and ShinyGO. The protein–protein interaction (PPI) was performed using the STRING database.

#### Plant-compounds network

The chemical analysis using LC–HR– ESI–MS technique led to the tentative identification of 18 compounds. From these, a basic network linking the plant (*A. graveolens* L.) to the identified compounds (18 compounds) was constructed.

#### Compounds-genes networks

A compounds-genes network was constructed based on chemical data informations obtained for each compound from the PubChem database (https://pubchem.ncbi.nlm.nih.gov/) [56] (last accessed on 03–05-2023) and Swiss Target Prediction database: http://www.swisstargetprediction.ch/result.php?job=215444691&organism=Homo_sapiens [57] (last accessed on 03–05-2023) was used to find out the targets of each identified compound related to the human species (Homosapien) was selected and the top targets were chosen in the Swiss Target Prediction database with a probability score > 0.

##### Genes-antiulcer network

DisGenet (https://www.disgenet.org/) [58] (last accessed on 04–05-2023) online database was used to find out the target genes related to gastric ulcer. Word filter option was used to focus on gene disease assosciations related to gastric ulcer.

##### Complete pharmacology network

The plants of the mixture were combined with the genes involved in peptic ulcer and identified types of gastric ulcer. This network and previously formed networks were constructed, visualized, and analyzed, using the software Cytoscape 3.9.0. (https://cytoscape.org/download.html) [59].

##### Protein–protein interaction network (PPI)

The interactions between proteins of genes annotated by *A. graveolens* L. metabolites and found to have a relation to gastric ulcer were established by the STRING database [60] (last accessed on 12–05-2023), the more function was applied.

##### Gene ontology and enrichment analysis

The gene ontology and enrichment analysis was performed to the all genes of the compounds under study involved in peptic ulcer to find out the GO terms of biological processes, cellular components, and molecular function that were affected by the annotated genes, using the ShinyGO 0.76 database (http://bioinformatics.sdstate.edu/go/), a graphical gene set enrichment tool [61] (accessed on 12–05-2023).

## Results

### Metabolomics profiling of the seeds of *Apium graveolens* L

**Fig. 2:**
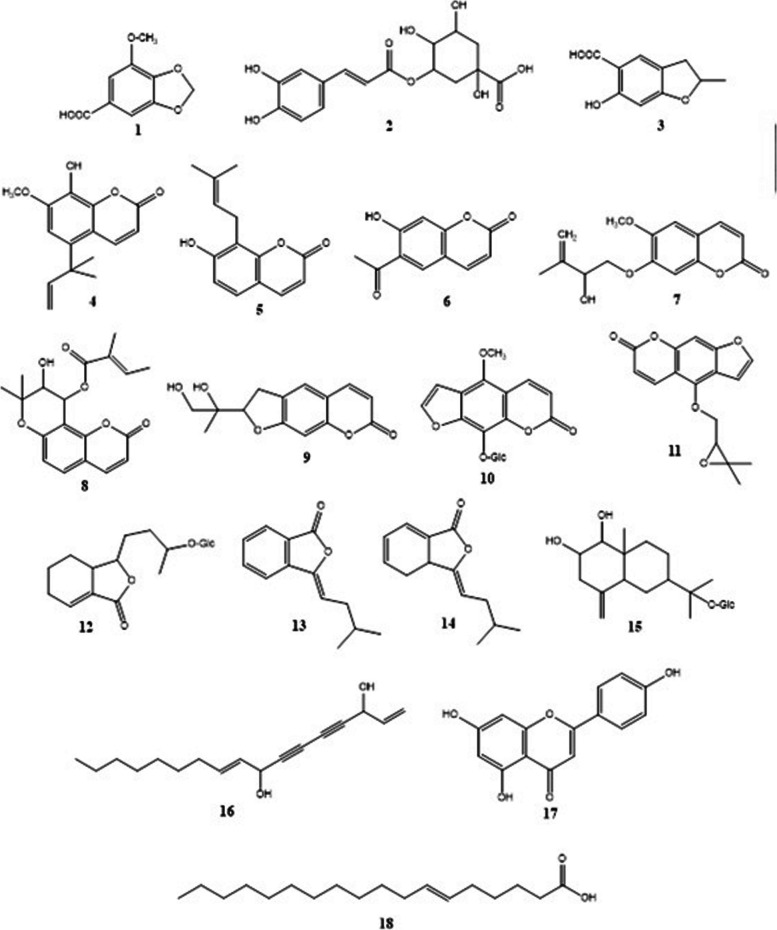
Chemical structures of the tentatively identified metabolites from *Apium graveolens* L. seeds

A set of secondary metabolites have been annotated resulting from the metabolomic analysis of *A. graveolens* seed extract utilizing HPLC, ESI, and HRMS followed by comparison with the DNP and METLIN databases. These secondary metabolites included phenolic acids, coumarins, furanocoumarins, phthalides, sesquiterpenes, polyacetylenes, flavonoids, and fatty acids (Table S1, Fig. 2). According to the obtained data, the mass ion peak at *m/z* 197.044 [M + H]^+^ for the suggested molecular formula C_9_H_8_O_5_ was identified as the phenolic acid, 3-methoxy-4,5-methylenedioxybenzoic acid (1), previously reported from the seeds of *A. graveolens* [62]. In addition, two more phenolic acids, namely chlorogenic acid (2) and 2,3-dihydro-6-hydroxy-2-methyl-5-benzofurancarboxylic acid (**3**), were dereplicated from the observed peaks at *m/z* 353.305 [M-H]^−^ and 195.066 [M + H]^+^, together with their corresponding molecular formulas C_16_H_18_O_9_ and C_10_H_10_O_4_, respectively. The former had previously been obtained from the leaves of *A. graveolens* [63], while the latter had been described before as one of the chemical constituents of *A. leptophyllum* seeds [64], but this is the first report of its detection in the seeds of celery. Furthermore, coumarins have been found as characteristic secondary metabolites of the genus *Apium*. In this vein, two coumarins with the molecular formulas C_15_H_16_O_4_ and C_14_H_14_O_3_ were characterized as celerin (4) and osthenol (5) based on the mass ion peaks at *m/z* 261.112 [M + H]^+^ and 231.101 [M + H]^+^, respectively. These compounds were reported among the formerly isolated phytoconstituents from *A. graveolens* seeds [38, 65, 66]. Another coumarin with the molecular formula C_11_H_8_O_4_ was characterized as 6-acetyl-7-hydroxy-2H-1-benzopyran-2-one (6), in line with the mass ion peak at *m/z* 203.033 [M-H]^−^; this molecule had been formerly described from *A. petroselinum* [67]. In addition, two more coumarins were identified as 7-(2-hydroxy-3-methyl-3-butenyloxy)-6-methoxycoumarin (**7)** and 10-tigloyloxy khellactone (8), in agreement with the observed peaks at *m/z* 275.091 [M-H]^−^ and 343.114 [M-H]^−^ and the predicted chemical formulas C_15_H_16_O_5_ and C_19_H_20_O_6_, respectively. Both molecules had been previously identified from the roots of some members of the Apiaceae family, namely *Bupleurum fruticosum* [68] and *Peucedanum japonicum* [69]. Of note, compounds 6‒8 are characterized herein for the first time.

Additionally, the current study revealed the presence of a number of furanocoumarins, represented by the compounds 9‒11. Of these, the mass ion peaks at *m/z* 263.128 [M + H]^+^, 395.331 [M + H]^+^, and 287.092 [M + H]^+^, in conformity with the predicted chemical formulas C_14_H_14_O_5_, C_18_H_18_O_10_, and C_16_H_14_O_5_ were described as 2,3-dihydro-2(1-hydroxy-1-hydroxy-methylethyl)-7H-furo[3,2g][1]benzopyran-7-one (9), 5-methoxy-8-O-β-D-glucosyloxypsoralen (10), and oxypeucedanin (11), respectively. The occurrence of compounds 9 and 10 were both in agreement with the findings of Garg et al., 1981 [70] and Ahluwalia et al., 1988 [71], who reported their presence in celery seeds, whereas compound 11, formerly obtained from *Peucedanum ostruthium* [72], was detected herein for the first time in the genus *Apium*.

Another group of compounds that have been widely reported as common secondary metabolites in various Apiaceae species, are the phthalides. In this context, celephthalide C (12) was dereplicated based on the mass ion peak at *m/z* 373.194 [M + H]^+^ , which was in line with the chemical formula C_18_H_28_O_8_. It had been obtained before from the seeds of *A. graveolens* [73]. In a similar way, two phthalides were annotated from the mass ion peaks at *m/z* 203.106 [M + H]^+^ and 205.123 [M + H]^+^, in consonance with the molecular formulas C_13_H_14_O_2_ and C_13_H_16_O_2_ and were characterized as 3-isovalidenephthalide (13) and 3-isovalidene-3a,4-dihydrophthalide (14), respectively; both are known metabolites of celery. Those were previously included as phytochemical ingredients, improving the flavor and aroma of *A. graveolens* leaves and stalks [38, 74].

In addition to the aforementioned metabolites, the mass ion peak at *m/z* 417.240 [M + H]^+^ was compatible with the suggested chemical formula C_21_H_36_O_8_ and was characterized as celerioside D (15). This glucosylated eudesmane-type sesquiterpenoids had previously been isolated from celery seeds [66], aerial parts, and roots [38]. Moreover, in accordance with the mass ion peak at *m/z* 261.185 [M + H]^+^ and the chemical formula C_17_H_24_O_2_, compound 16 was identified as falcarindiol, a common polyacetylene that had earlier been reported from the roots of celery [75]. Similarly, the flavone luteolin (17) was identified from the observed peak at *m/z* 287.237 [M + H]^+^, together with its corresponding molecular formula C_15_H_10_O_6_. This flavone had previously been derived from the leaves of *A. graveolens* [76]. On the other hand, the mass ion peak at *m/z* 281.247 [M-H]^−^ for the predicted molecular formula C_18_H_34_O_2_ was identified as 6-octadecenoic acid (18). This molecule had been detected before in celery seed oil [77].

### In vivo antiulcer activity

#### Effect of *A. graveolens* extract on the ulcer index of rats treated with indomethacin

**Fig. 3:**
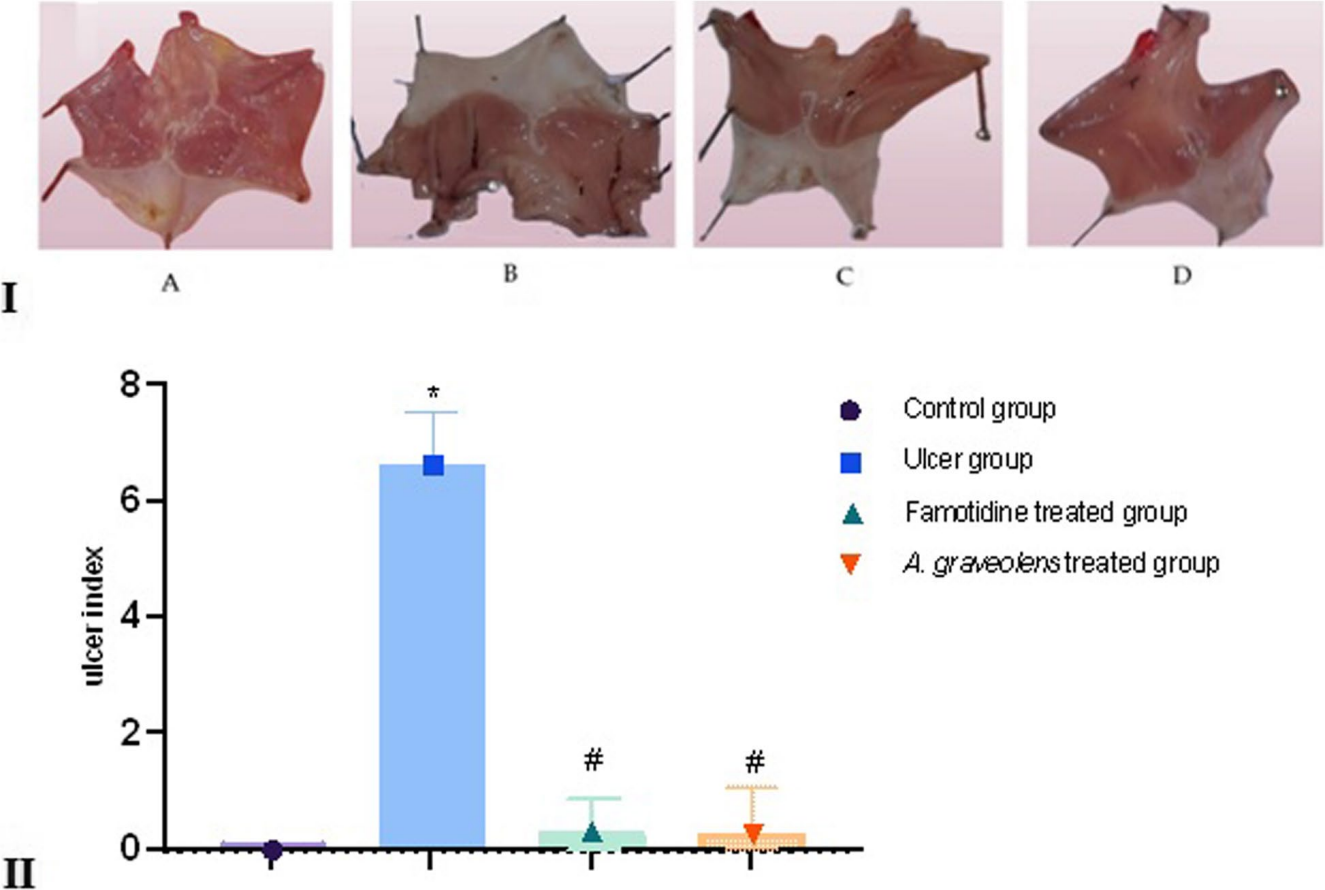
(I): Photomacrographs of the stomach of a representative rat, which have been cut along the greater curvature. A represents the control group treated with a vehicle substance. B represents the experimental group treated with indomethacin. C represents the experimental group treated with famotidine. D represents the experimental group treated with *A. graveolens* extract. The presence of both circular and linear gastric ulcers was observed in the indomethacin-treated group, which were significantly reduced by the administration of the reference drug (famotidine) and the *A. graveolens* extract. (II): The impact of indomethacin, both in isolation and in conjunction with pre-treatments of famotidine, as well as the *A. graveolens* extract, on the gastric ulcer index in rats. The statistical analyses were conducted employing ANOVA followed by Tukey's post hoc test. The sample size was *n* = 6, and the results are presented as the mean ± SEM. * Significantly different from the control group at *p* ≤ 0.05. # Significantly different from the indomethacin-alone group (ulcer group) at *p* ≤ 0.05

The macroscopic analysis of the stomachs obtained from the control group revealed the presence of a typical and healthy pink hue in the gastric mucosa. The folding pattern appeared normal, and the mucosal layer displayed a regular thickening, without any observable signs of inflammation or ulceration. The stomachs that were isolated from the indomethacin group exhibited pronounced congestion, along with longitudinal irregular mucosal lesions of varying diameters and depth, which were dispersed throughout the entirety of the gastric surface. Stomachs that were isolated from various treatment groups exhibited mild congestion and mild hemorrhagic mucosal lesions, suggesting the presence of preventive effects for both the reference drug (famotidine) and the tested extract. The administration of indomethacin resulted in a notable elevation in the ulcer index (6.63) when compared to the control group (*p* ≤ 0.05). In contrast, the extract derived from *A. graveolens* exhibited a notable reduction in the ulcer index (0.28) when compared to the indomethacin group (*p* ≤ 0.05). These effects were found to be comparable to the reported effects of famotidine (0.3). It is noteworthy to mention that there was no discernible disparity observed between the control group and the experimental groups subjected to famotidine or *A. graveolens* treatment. The findings are depicted in Fig. 3.

#### Histopathological study

**Fig. 4:**
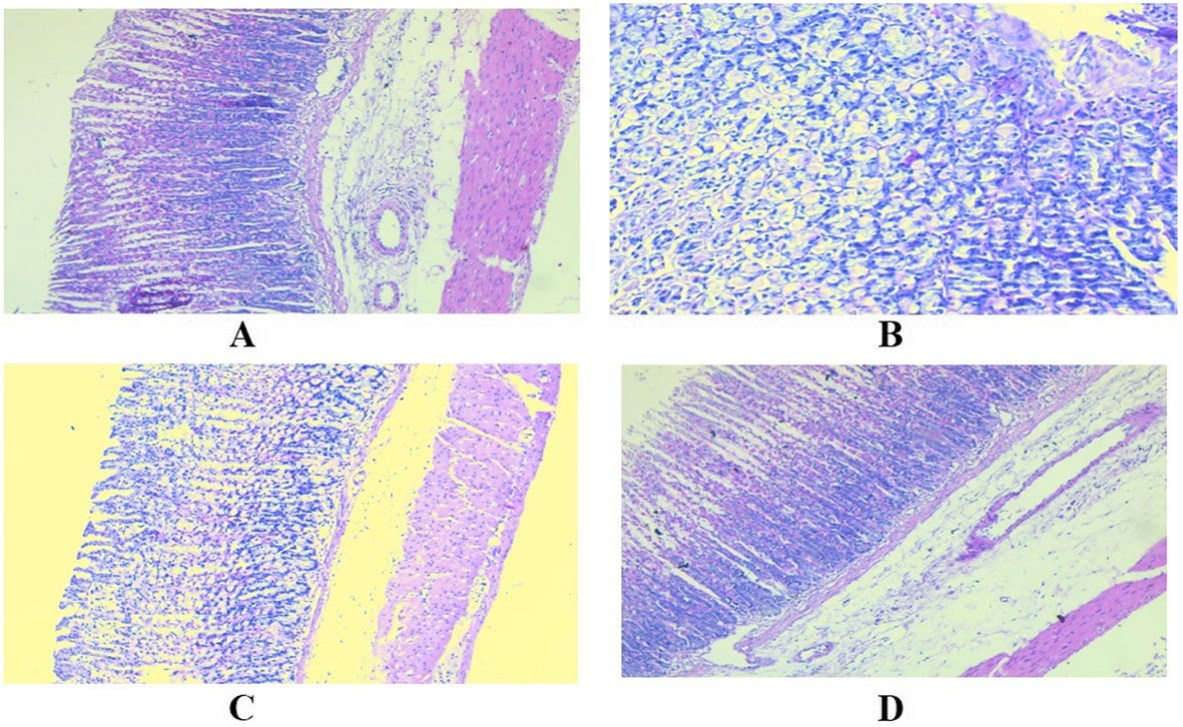
**A** Section in the control group showing normal gastric mucosa showing normal surface mucous cells and normal parietal cells; **B** section in the ulcer group exhibiting a wide area of coagulative necrosis in the gastric mucosa; **C** section within the group treated with famotidine exhibited significant reductions in the degenerative and necrotic alterations observed in the gastric mucosa; **D** section in the *A. graveolens* treated group revealing complete resolution of gastric ulcer with restoration of normal mucosa and absence of coagulative necrosis, hematoxylin and eosin staining (H&E $\times$ 100 and 200)

Indomethacin administration resulted in significant gastric damage, as demonstrated by the presence of severe degrees of comparative necrotic changes within the gastric mucosa, along with the infiltration of inflammatory cells, according to a histopathological analysis of the stomachs of the various rat groups. The pre-treatment with famotidine significantly reduced the degenerative and necrotic alterations inside the gastric mucosa, indicating a clear mitigation of the pathological changes in the stomachs. By preventing gastric inflammation inside the surface mucosal membrane of the stomach, pre-treatment of rats with *A. graveolens* led to a significant reduction of the degenerative alterations within the stomach glands (Fig. 4).

#### The impact of *A. graveolens* extract on changes in gastric acidity caused by indomethacin in the gastric mucosa of rats

**Fig. 5:**
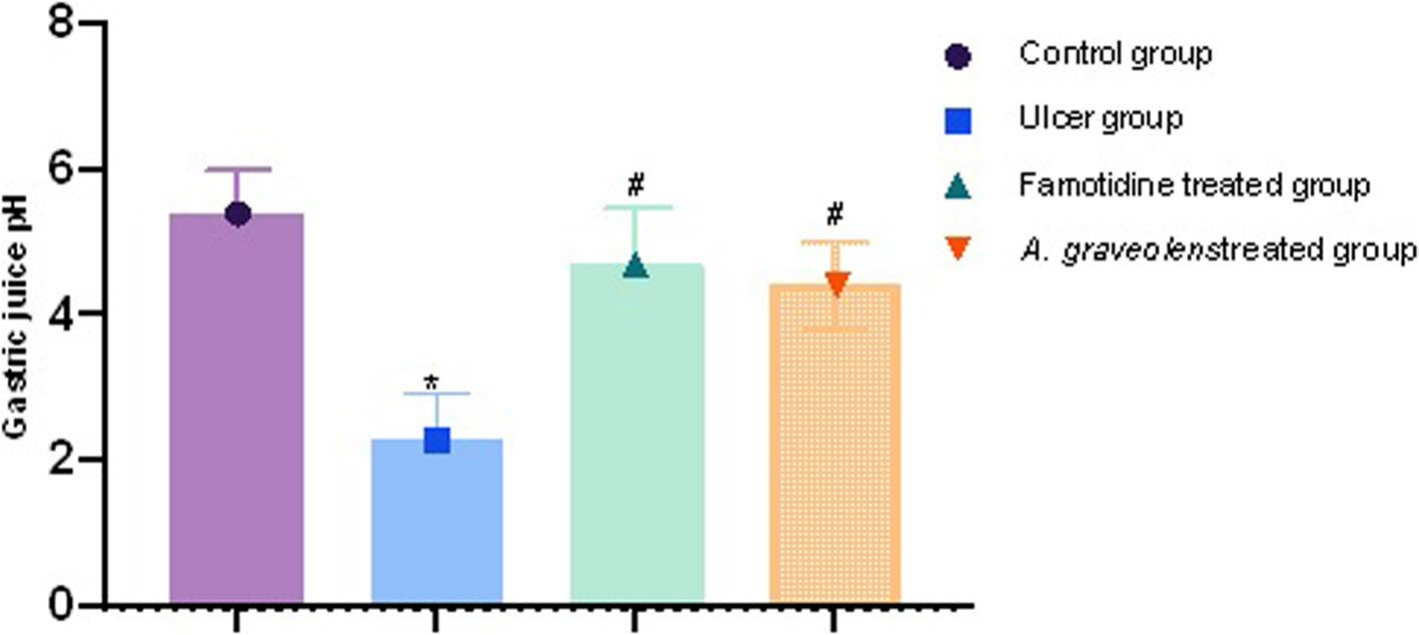
The impact of indomethacin, either in isolation or in conjunction with oral pre-treatments of famotidine or *A. graveolens* extract, on the gastric pH of rats was investigated. The statistical analyses were performed employing one-way ANOVA followed by Tukey's post hoc test. The sample size was *n* = 6, and the results are presented as the mean ± SEM. * Significantly different from the control group at *p* ≤ 0.05. # Significantly different from the indomethacin-alone group at *p* ≤ 0.05

The gastric acidity exhibited an elevation, as evidenced by the notable decrease in gastric pH observed in the rats treated with indomethacin (2.3), in comparison to the control group (Fig. 4, *p* ≤ 0.05). The administration of famotidine (4.7) or Apium (4.4) resulted in a notable reduction in gastric acidity when compared to the indomethacin group (*p* ≤ 0.05). However, there was no significant distinction observed between the groups treated with famotidine and *A. graveolens* extract (Fig. 5, *p* ≤ 0.05).

#### Effects of *A. graveolens* extract on changes in oxidative stress markers induced by indomethacin in rat gastric mucosa

**Fig. 6:**
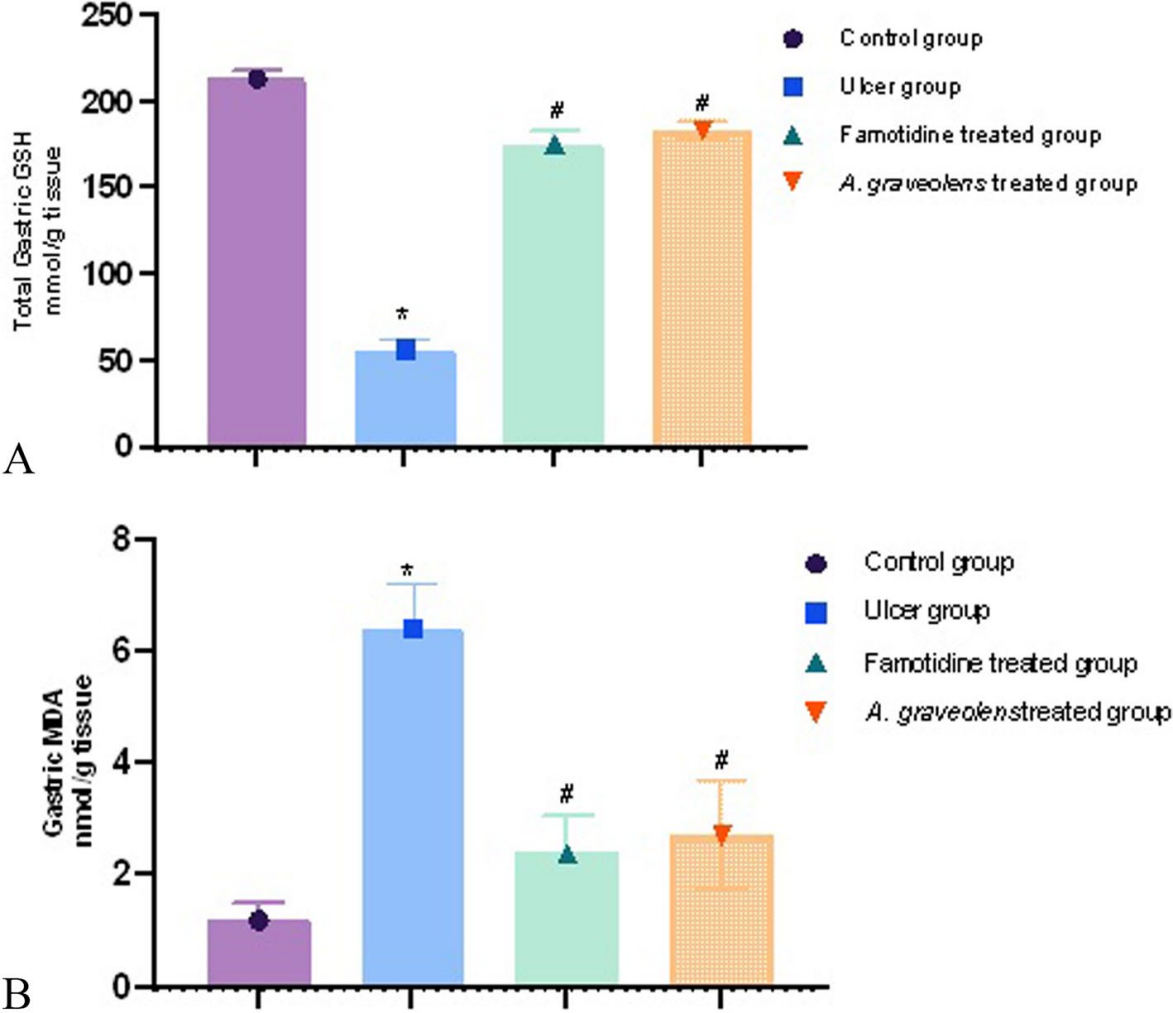
The impact of indomethacin in isolation and in combination with orally administered famotidine or *A. graveolens* extract on **A** gastric reduced GSH levels and **B** MDA concentrations in rats was investigated. The statistical analyses were conducted using a one-way ANOVA followed by Tukey's post hoc test. The sample size was *n* = 6, and the results are presented as the mean ± SEM. * The experimental group exhibited a statistically significant difference compared to the control group, with a *p*-value ≤ 0.05. # The experimental group exhibited a statistically significant difference compared to the indomethacin-alone group, with a *p*-value ≤ 0.05

In order to elucidate the gastroprotective mechanism of the extract, we conducted additional analysis on oxidative stress markers present in the gastric mucosa. Our findings revealed that the administration of indomethacin resulted in a significant reduction in gastric GSH levels (56.9 mmol/g tissue) when compared to the control group (*p* ≤ 0.05). Rats subjected to pre-treatment with famotidine or *A. graveolens* extract, resulting in an observed elevation of gastric GSH levels measuring 175.3 and 183.5 mmol/g tissue, respectively (*p* ≤ 0.05, as depicted in Fig. 6). In contrast, the administration of indomethacin resulted in a notable elevation in the concentration of MDA by 6.4 units within the gastric tissues (*p* ≤ 0.05) when compared to the control group. Both famotidine and *Apium* exhibited a notable reduction in MDA levels (2.4 and 2.71, respectively) (*p* ≤ 0.05) when compared to the indomethacin group (Fig. 6).

#### Effect of *A. graveolens* on gene expression of inflammatory cytokines

**Fig. 7:**
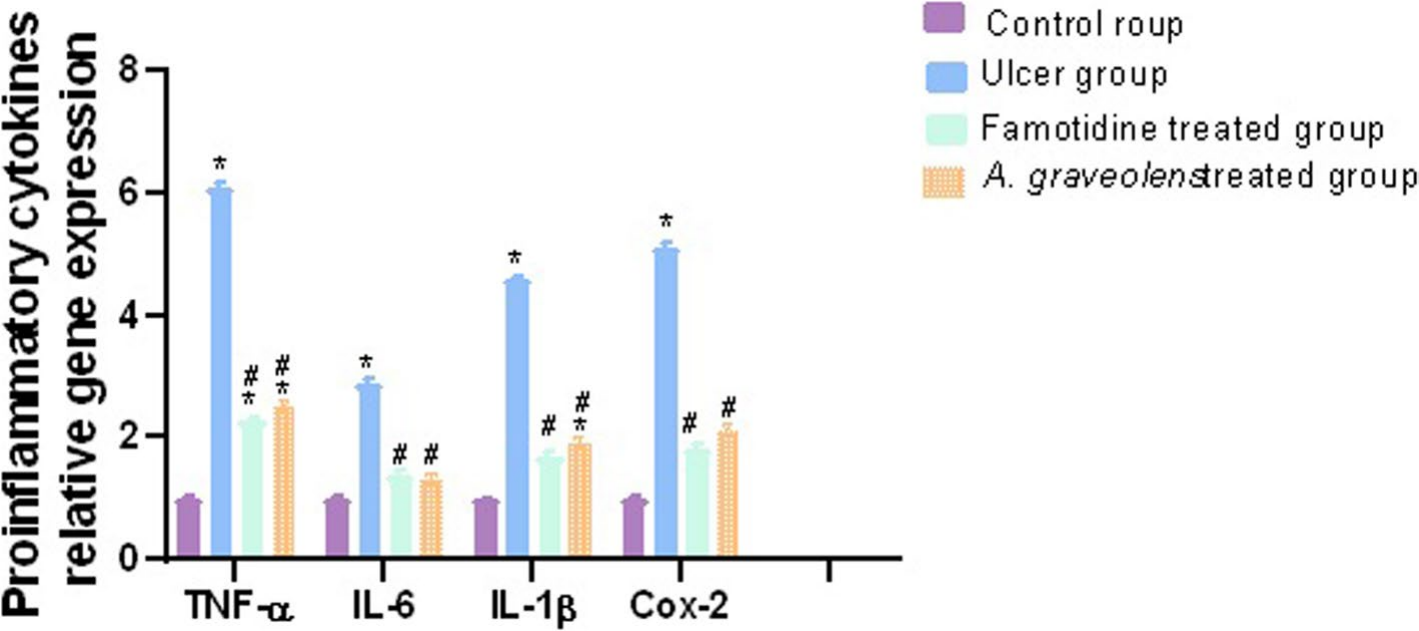
Relative expression of pro-inflammatory cytokine TNF-a, IL-6, IL-1β, and COX-2 were determined in all the groups under investigation using the qRT-PCR technique. Following normalization to GAPDH, the data were displayed as fold change compared to the control group. The bars reflect the mean ± SEM, *n* = 6. A one-way ANOVA test followed by Tukey’s post hoc test was utilized to ascertain the notable distinction among the various groups, with * *p* ≤ 0.05 in comparison to the control group and # *p* ≤ 0.05 compared to the indomethacin group

The administration of indomethacin resulted in the development of gastric inflammation, which was evident through significant increases in the levels of TNF-α (6.1-fold), IL-6 (2.9-fold), IL-Iβ (4.6-fold) and COX-2 (5.1-fold) when compared to the control group (*p* ≤ 0.05). The administration of *A. graveolens* extract resulted in a substantial decline (*p* ≤ 0.05) in the elevated levels of TNF-α (2.5-fold), IL-6 (1.3-fold), IL-1β (1.9-fold), and COX-2 (2.1-fold) when compared to the group treated with indomethacin. In a similar manner, famotidine significantly decreased the inflammatory markers to TNF-α (2.3-fold), IL-6 (1.4-fold), IL-1β (1.7-fold) and COX-2 (1.8-fold) versus the indomethacin group (Fig. 7).

#### Effect of *A. graveolens* on gene expression of inducible nitric oxide synthase (iNOS)

**Fig. 8:**
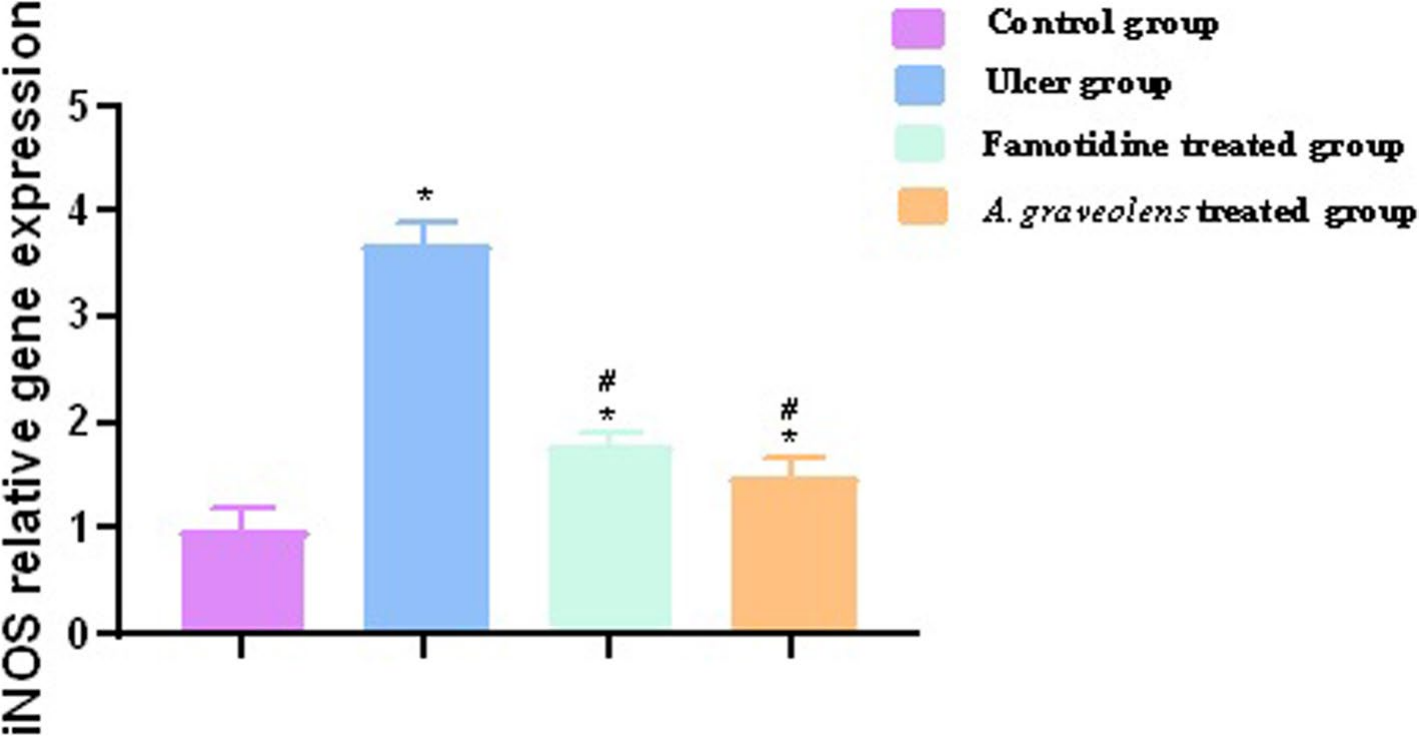
Relative expression of iNOS in all study groups was assessed by real-time PCR. Following normalization to GAPDH, the data were displayed as fold change compared to the control group. The bars reflect the mean ± SEM, *n* = 6. A one-way ANOVA test followed by Tukey’s post hoc test was employed to ascertain the notable disparity among the various experimental groups in the study, with * *p* ≤ 0.05 comparing to the control group and # *p* ≤ 0.05 compared to the indomethacin group

The gene expression of iNOS was assessed in the gastric tissues acquired from the rat model with gastric ulcers induced by indomethacin. Within the group treated with indomethacin, a discernible upregulation of iNOS was observed in the gastric tissues when compared to the control group. Rats subjected to treatment with *A. graveolens* or famotidine exhibited a notable reduction in the expression of iNOS within the gastric tissues, which was statistically significant in comparison to the group treated with indomethacin (*p* ≤ 0.05) (Fig. 8).

#### Effect of *A. graveolens* on NF-κB P65 and VEGF

**Fig. 9:**
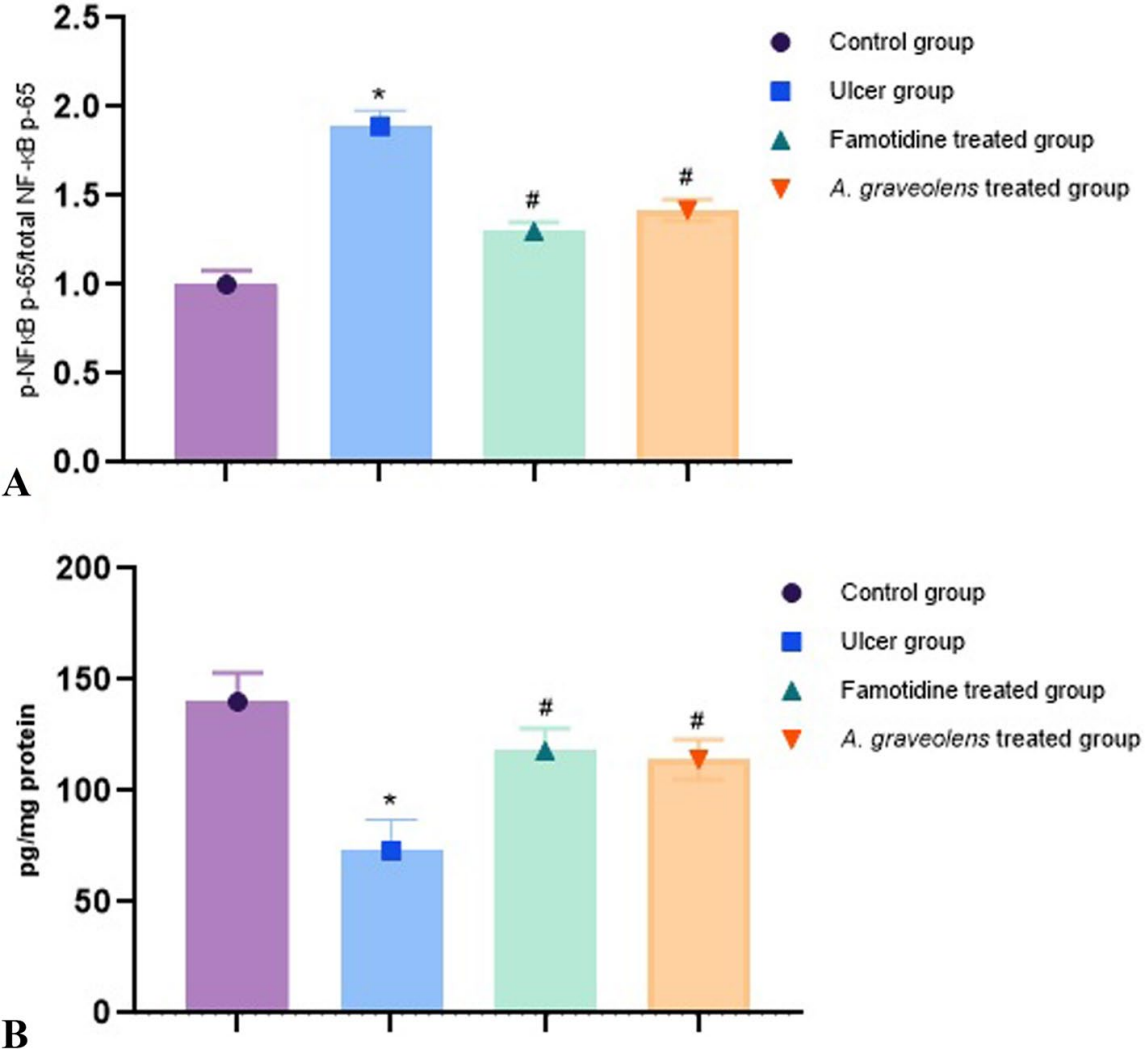
Effect of *Apium* extract on: **A** p-NF-κB p65 (Ser536), in the indomethacin-induced gastric lesions rat model; **B** Effect of *Apium* extract on gastric VEGF level in the indomethacin-induced gastric lesions rat model. Data are presented as the mean ± SEM (*n* = 6 per group; one-way ANOVA followed by Tukey’s multiple comparison test; with * *p* ≤ 0.05 comparing to the control group and # *p* ≤ 0.05 compared to the indomethacin group

Then, we detected the expression levels of the classical inflammatory pathway NF-κB in the gastric tissues. As shown in Fig. 9 A, indomethacin significantly elevated the protein expression levels of p-NF-κB p65/total NF-κB p65 (1.89-fold) compared to the control group. *A. graveolens* extract significantly inhibited the indomethacin induced p-NF-κB p65/total NF-κB p65 (1.42-fold) protein expression levels. Similarly, famotidine markedly diminished the elevated levels of p-NF-κB p65/total NF-κB p65 (1.3-fold) proteins versus the indomethacin group (*p* ≤ 0.05).

Subsequently, the expression VEGF in the gastric tissues was estimated using the ELISA assay. Indomethacin significantly decreased the protein expression levels of VEGF (73.1 pg/mg protein) compared to the control group. *A. graveolens* extract significantly restored VEGF protein expression levels compared with the indomethacin group (114.6- pg/mg protein). Similarly, famotidine markedly elevated the levels of VEGF in the stomach (118.7 pg/mg protein) versus the indomethacin group (*p* ≤ 0.05) (Fig. 9B).

### Network pharmacology study

#### Plant-metabolite network

**Fig. 10:**
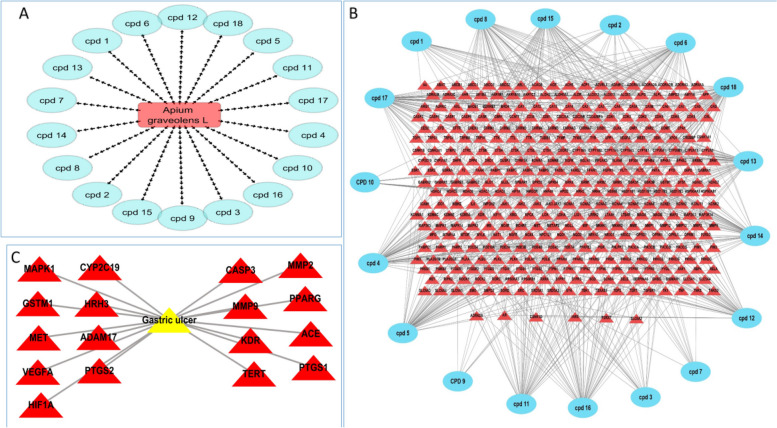
**A** plant-metabolite, **B** metabolite-gene, **C** gene-peptic ulcer; the pink rectangle is the *A. graveolens* extract, the blue oval shapes represent metabolites, red triangles are the annotated genes, and the yellow triangle symbolizes the peptic ulcer disease

The active metabolites were identified from *A. graveolens* L. seeds using LC–HR– ESI–MS. The identified 18 metabolites were connected to the plant in a simple network (plant-metabolites) (Fig. 10A).

#### Metabolite-gene network

The identified metabolites are associated with 379 unique genes, the formed network (metabolite-gene) consisted of 397 nodes represent identified metabolites and annotated genes, and 672 edges, with characteristic path length of 3.605 and network centralization of 0.134 (Fig. 10B).

#### Gene-gastric ulcer network

Based on the DisGeNET database, the gene-disease associations were obtained, the whole gene-disease associations were 48,712, using the filter option in the database was utilized to focus on non-malignant gastric disorders assembled in form of 90 gene-disease associations for 58 unique genes. In order to focus on the gastric ulcer condition, further filtration was performed using filter word (built in option in the DisGeNET database), which resulted in the determination of 17 genes among the studied data set related to gastric ulcer (Fig. 10C).

#### Complete pharmacology network

**Fig. 11:**
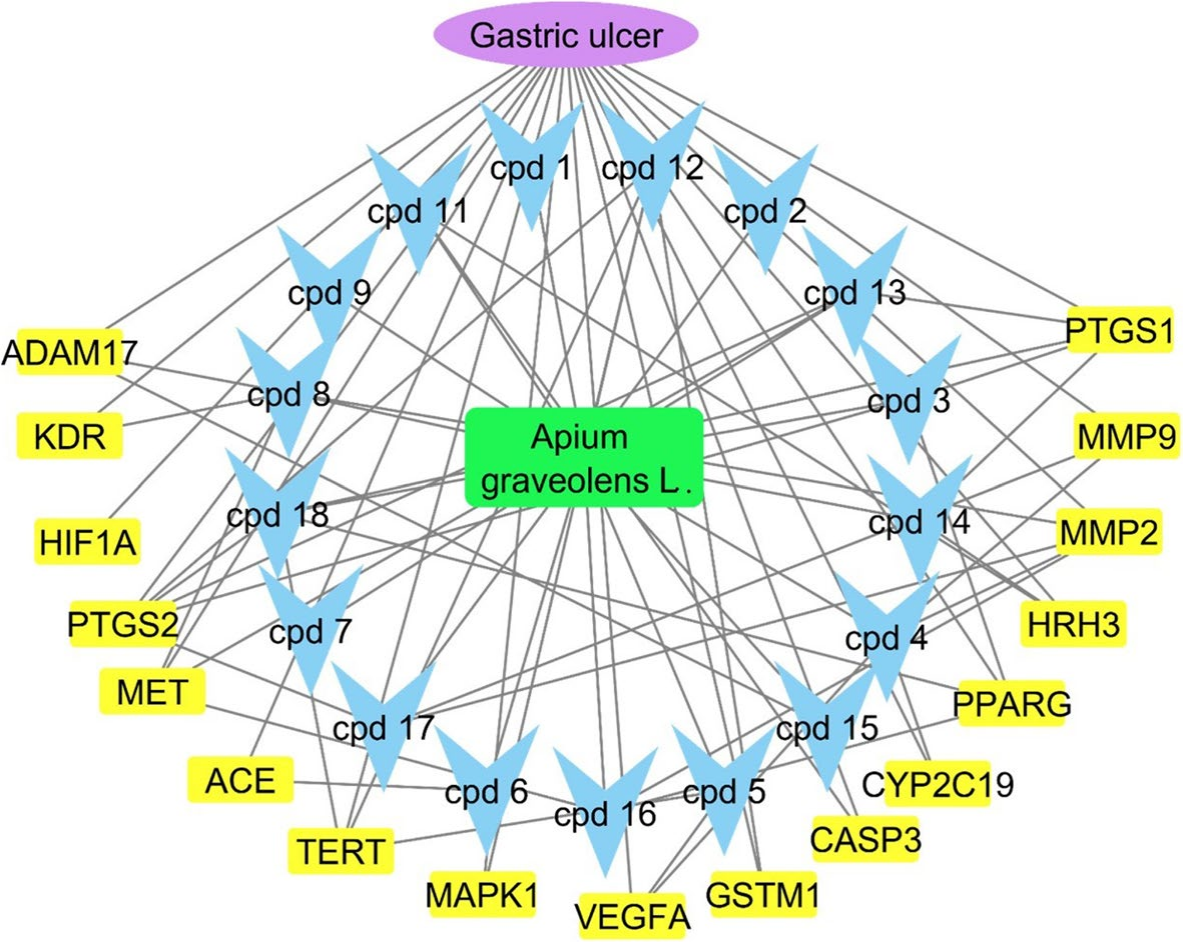
Complete pharmacology network: the green rectangle symbolizes the plant, blue arrow heads represent the identified metabolites, yellow rectangles are the annotated genes with relation to gastric ulcer

The complete pharmacology network was formed using the extracted data in a reverse manner to connect the gastric ulcer to corresponding annotated genes and connected these genes to their corresponding identified metabolites. This network was formed through merging the previously formed networks and excluding the genes not related to gastric ulcer to simplify the complete pharmacology network. The formed network consisted of 36 nodes and 70 edges and characteristic path length of 2.321 and network centralization of 0.397 (Fig. 11). Compounds 4, 8, 12, and 18 are represented by four edges in the network for each, which means that each of these compounds is linked to four genes related to gastric ulcer. PTGS2 was the top identified gene with six edges, followed by MMP2 and PTGS1 with five edges for each.

##### Protein–protein interactions

**Fig. 12:**
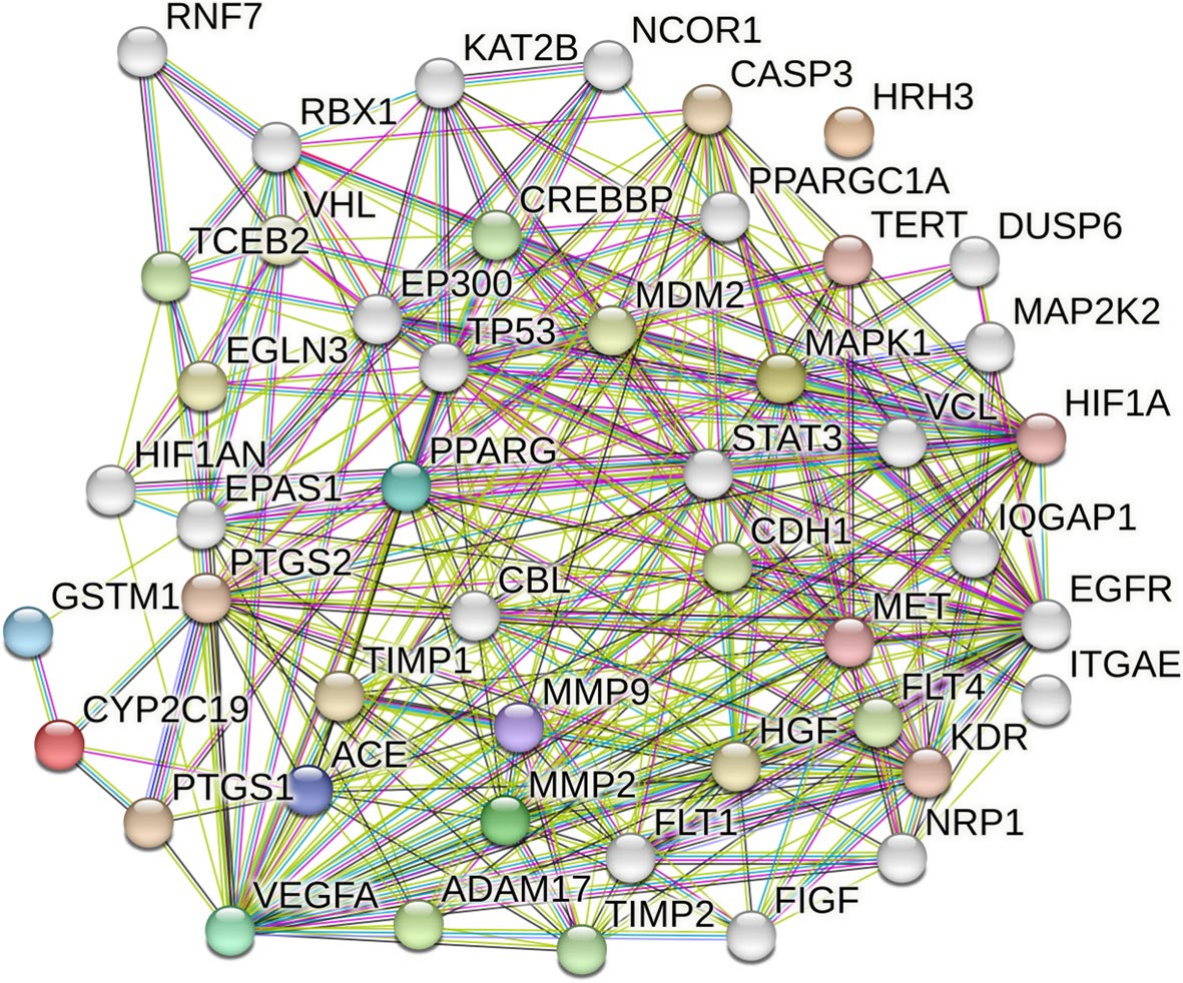
Protein–protein interactions of the 17 genes identified by *Apium graveolens* L. metabolites correlated to gastric ulcer

The analysis of the interactions between the proteins of the genes related to gastric ulcer was performed using the STRING database, the interactions between the proteins of the 17 genes was performed using three more functions, the formed network consisted of 47 nodes and 362 edges, with an average local clustering coefficient of 0.683. The PPI network identified VEGFA, HIF1A, TP53, and EGFR as the top genes according to node degree in descending order (Fig. 12).

##### Gene enrichment analysis

**Fig. 13:**
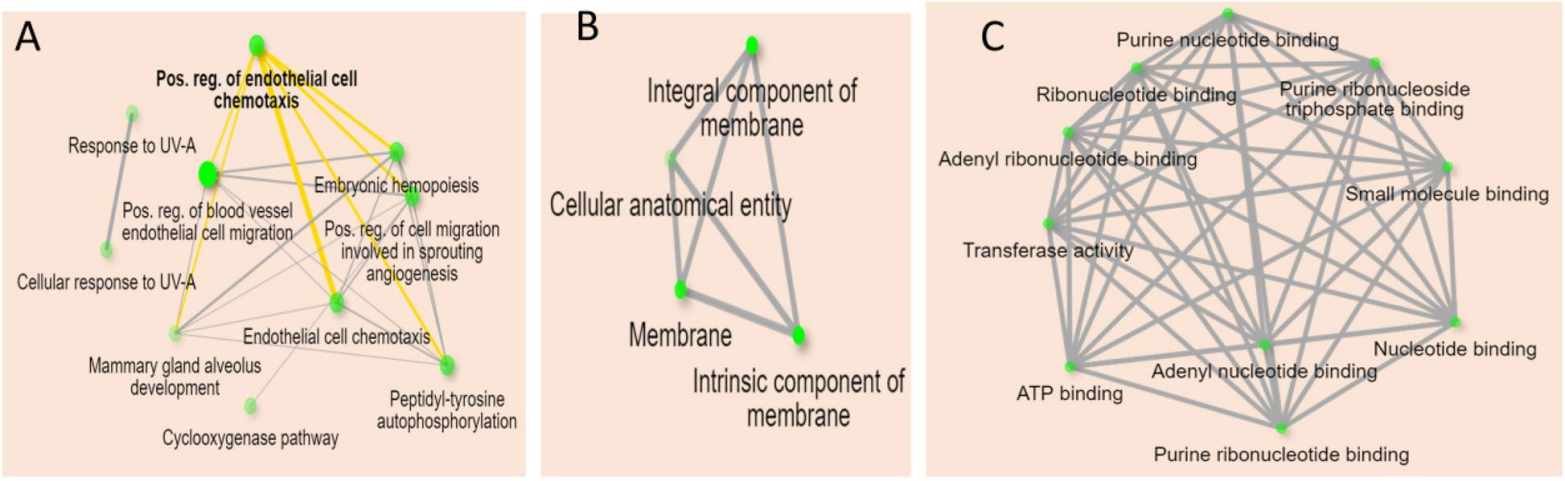
Networks describing the top GO analysis concerning **A** biological process; **B** cellular component; **C** molecular function

**Fig. 14:**
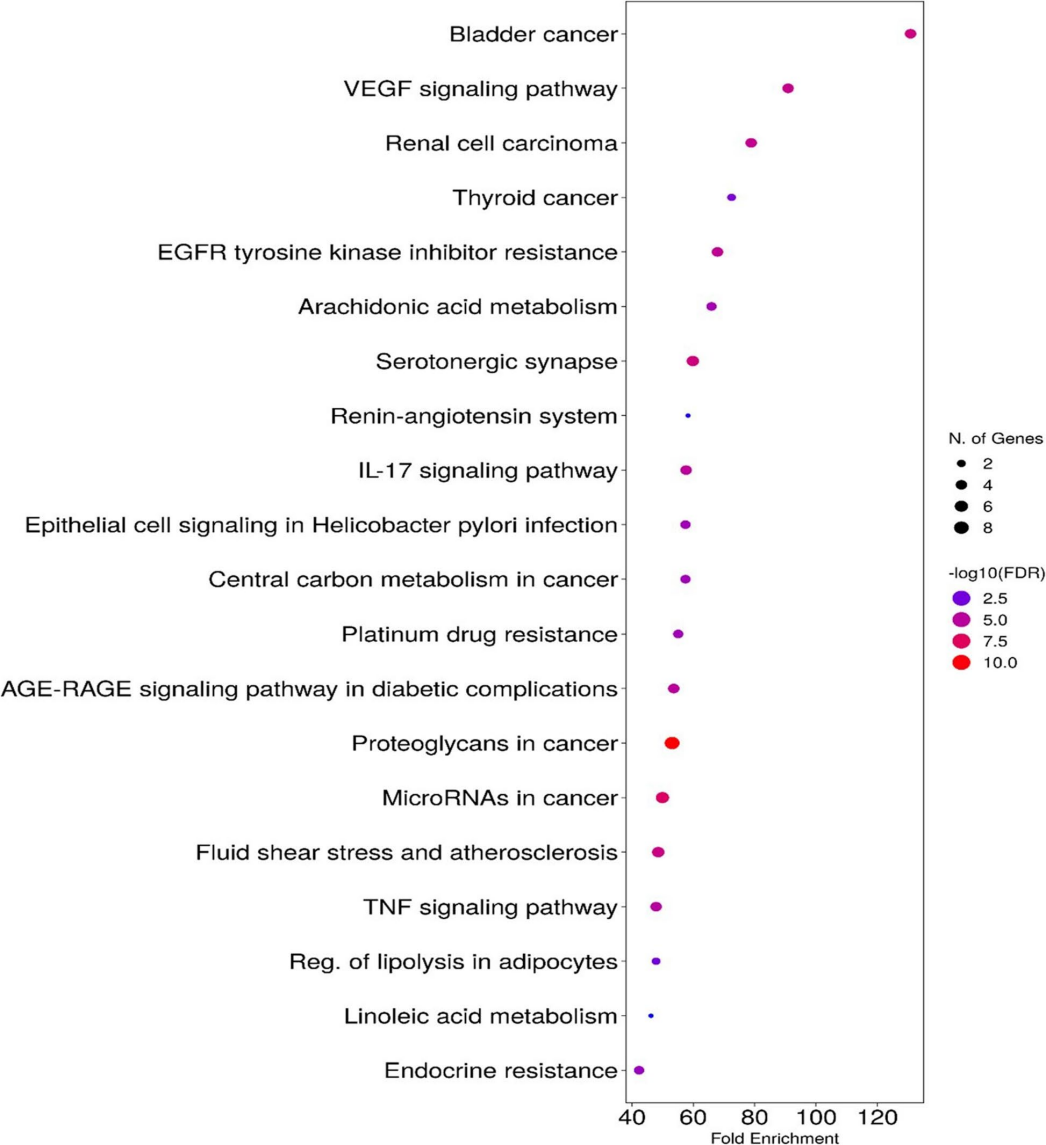
Top 20 biological KEGG pathways identified by the 17 genes identified by *Apium graveolens* L. related to gastric ulcer

**Fig. 15:**
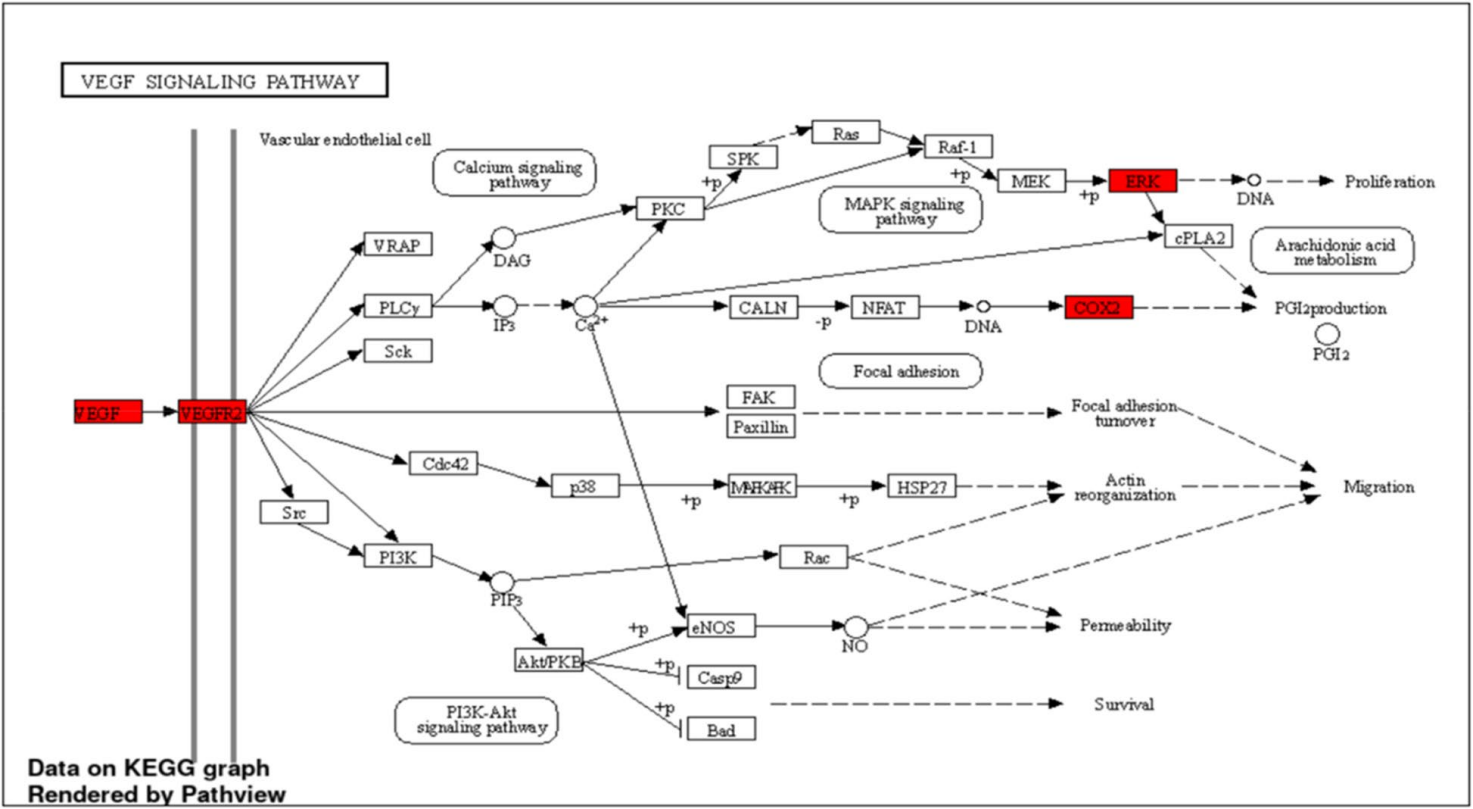
The top signaling pathway (VEGF signaling pathway), the genes among the studied data of the anti-gastric ulcer are highlighted in red [78]

The gene enrichment analysis was performed for the 17 genes related to the gastric ulcer to focus on the possible gene enrichment analysis towards gastric ulcer. The gene enrichment was detected in terms of biological process, cellular component, and molecular function in addition to biological pathways. Each term was classified according to fold enrichment in descending order (Tables S2 and S3), the top biological process is a positive regulation of endothelial cell chemotaxis (Fig. 13A). The top 2 cellular components are equal in fold enrichment; integral component of membrane and intrinsic membrane component (Fig. 13B). The top 10 molecular functions are equal in fold enrichment (Fig. 13C). The biological pathways from the KEGG database identified and visualized by ShinyGO database are 154 pathways. The top KEGG biological pathway according to fold enrichment is the bladder cancer and the top signaling pathway is the VEGF signaling pathway (Figs. 14 and 15).

## Discussion

Gastric ulcers continue to be a prevalent gastrointestinal disorder on a global scale. Indomethacin exhibits a greater propensity for ulcerogenesis compared to other NSAIDs. Henceforth, it is regarded as the preferred pharmaceutical agent for instigating harm to the gastric mucosa within an experimental milieu [79]. The ingestion of NSAIDs leads to an increased ulcer index, as evidenced by the presence of distinct long hemorrhagic bands and petechial lesions with ulcerative inflammation [80], Additionally, there is observed gastric degeneration and necrosis [81], along with the death of epithelial cells and the occurrence of local or multiple hemorrhagic ulcers [82]. The observed phenomenon can be ascribed to the upregulation of diverse inflammatory cytokines and chemokines that possess chemotactic properties towards leukocytes and other cells involved in inflammatory responses [83]. Additionally, it can be partially attributed to the induction of oxidative stress [80].

Pre-treatment with the seed extract of *A. graveolens* exhibited a notable amelioration of gastric mucosal damage, as indicated by a significant decrease in the ulcer index, comparable to the preventive effect observed with famotidine (the reference drug). Our observations exhibited similarity to the outcomes of previous investigations, which have likewise exhibited the manifestation of gastric mucosal damage induced by indomethacin [84, 85].

Stimulation of gastric mucosal damage occurs through the activation of ROS and cytokines, both through direct and indirect mechanisms [86]. Experimental investigations have provided evidence suggesting that ROS play a significant role in the development of gastric ulcers induced by NSAIDs [87]. The metabolism of NSAIDs through the action of peroxidase enzymes is accountable for the pro-oxidant properties exhibited by indomethacin in the gastric mucosa [88]. Elevated levels of ROS have been observed to be linked with heightened lipid peroxidation of the membranes of gastric cells. This phenomenon is also accompanied by an increase in mucosal MDA levels, a decrease in the secretion of gastric mucus, and the occurrence of DNA damage [89]. MDA is widely recognized as a biomarker that is intricately associated with the process of lipid peroxidation. Fascinatingly, within our research, the heightened concentration of MDA, which is linked to damage in the mucosal tissue, exhibited a significant decrease upon administration of *A. graveolens* seed extract. In contrast, GSH plays a pivotal role as one of the primary cellular antioxidant mechanisms, ensuring the maintenance of the cell redox status [90, 91]. The levels of GSH in gastric tissues exhibited a notable reduction subsequent to the administration of indomethacin. The observed decrease in GSH content could potentially be attributed to the generation of superoxide radicals, as these radicals have a tendency to deplete a significant quantity of endogenous antioxidant enzymes during scavenging [82, 92]. The exacerbation of gastric mucosal damage can be attributed to the declined level of GSH, which leads to a rapid increase in hydrogen peroxide and lipid peroxides within the cells of the gastric mucosa. Hence, an increase in the expression of antioxidant enzymes and a rise in the gastric content of GSH may serve as a significant protective mechanism against gastric ulcers that are linked to oxidative stress [93].

The remarkable gastroprotective activity of *A. graveolens* seed extract might be attributed to its rich chemical profile, which was deduced through metabolomics profiling analysis using LC-HR-ESI–MS. This has led to the tentative identification of 18 different compounds. These constituents actually belong to various phytochemical classes, including coumarins, phenolic acids, and flavonoids, previously reported to have potent anti-inflammatory and antioxidant effects [94–96]. They preserve the gastric GSH level by acting as scavenging free radicals instead of GSH and preventing lipid peroxidation, which can ultimately accelerate the healing of ulcers. Interestingly, phenolic acids as well as coumarins have also been shown to exhibit anti-ulcer and gastroprotective effects on experimental gastric acid ulcer models in rats [97, 98]. These metabolites can protect the gastric mucosa via several mechanisms of action, such as enhanced mucus production, antioxidant and free-radical scavenging properties, antisecretory action, prevention of *Helicobacter pylori* growth, and stimulation of the antioxidative defense enzyme activities [99].

Gastric ulcer repair necessitates the restoration of epithelial structures and the underlying connective tissue, which involves cell proliferation and angiogenesis. Several growth factors have been linked to the healing of ulcers [100]. VEGF is a key angiogenic element that promotes the creation of granulation tissue and new micro capillaries via angiogenesis, which speeds up the healing of gastric and duodenal ulcers [101]. In our research, we discovered that indomethacin dramatically reduced VEGF expression in gastric tissues. *A. graveolens* treated rats, on the contrary, have significantly enhanced VEGF expression in gastric regions, aiding the healing process.

NF-κB, a key transcription factor, exerts significant influence over various immune and inflammatory mechanisms, encompassing the intricate orchestration of gastric ulcer formation [102]. NF-κB exhibits swift production upon cellular injury, encompassing the exposure to ROS. The initiation of NF-κB activation occurs through the degradation of the IκB-α protein, which is triggered by signals. The NF-κB complex, once activated, undergoes translocation from the cytoplasmic region to the nucleus. This migration enables the complex to generate transcription factors like TNF-α, IL-1β, and IL-6 [79, 103]. The present study has demonstrated that the induction of gastric injury triggers the activation of the IKκB/NF-κB signal transduction pathway. This is evident from the significant elevation in levels of phosphorylated NF-κB p-65 protein in the gastric mucosa, when compared to the mucosa of rats in the control group. The extract exhibited significant reduction in the IKκB/NF-κB signaling pathway activated by indomethacin. This was evident from the notable decrease in levels of phosphorylated NF-κB p-65 protein in comparison to the ulcer group. The extract exerted inhibitory effects on the activation of NF-κB by impeding the phosphorylation process and subsequent degradation of IκB-α. Previous research has demonstrated that antioxidants, including polyphenolics, impede the activation of NF-κB by inhibiting the phosphorylation of IκB-α induced by signaling [104]. The observed decrease in NF-κB p-65 concentration in rats pretreated with *A. graveolens* could potentially be attributed to the plant extract's capacity to scavenge ROS [105]. Additionally, it has been documented that the inhibition of NF-κB p65 hinders the expression of COX-2, a pro-inflammatory mediator involved in gastrointestinal damage [106]. The inhibitory effects of *A. graveolens* seed extract on COX-2 expression were observed, suggesting a potential correlation with the extract's gastroprotective properties. In this context, the inhibitory impacts of the botanical extract on the IKκB/NF-κB signaling pathway could potentially elucidate the reduced expression of COX-2 and the mitigation of gastric inflammation induced by indomethacin [107].

Inflammatory cytokines, including TNF-α, IL-1β, and IL-6, are secreted as part of the immune response, resulting in the attraction of neutrophils and mononuclear cells to the lamina propria. In accordance with scientific observations, heightened levels of TNF-α, IL-1β, and IL-6 have been found to exhibit a clinical correlation with the magnitude of gastric inflammation. Having confirmed that numerous biological agents able to diminish the levels of pro-inflammatory cytokines such as TNF-α, IL-1β, and IL-6, could potentially exhibit effectiveness in the management of gastric ulceration [108]. In this context, indomethacin induced a pronounced inflammatory response in the gastric mucosa, as evidenced by notable increases in the infiltration of inflammatory cells, along with elevated levels of TNF-α, IL-6, and IL-1β, when compared to the undisturbed gastric mucosa observed in the control rats. The suppressive effects of the *A. graveolens* extract on the IKκB/NF-κB signaling pathway may be linked to its ability to reduce the levels of IL-1β, IL-6, and TNF-α, as well as its antioxidant properties [107, 109]. The tested extract exhibited the ability to inhibit the infiltration of inflammatory cells in the gastric region, leading to significant reductions in the levels of TNF-α, IL-1β, and IL-6 within the mucosal tissue. These findings suggest that *A. graveolens* plays a role in mitigating inflammation. These findings align with prior observations indicating that an excessive production of pro-inflammatory cytokines is causative of gastric mucosal injury and exacerbates the inflammation of the gastric mucosa [79]. Therefore, by reducing TNF-α, IL-6, and IL-1β expression, *A. graveolens* may be considered as an alternative agent for treating gastric ulcer patients.

IL-6 exerts its stimulatory effects on lymphocytes, macrophages, and neutrophils present at the site of inflammation. This stimulation subsequently triggers an overproduction of ROS and lysosomal enzymes. Consequently, these excessive levels of ROS and lysosomal enzymes contribute to the detrimental tissue damage observed in gastric ulcers. TNF-α exerts a significant influence on the development of gastric mucosal injury caused by indomethacin [110]. This influential cytokine diminishes blood flow to the gastric mucosa and enhances the expression of gastrin and vascular endothelial growth factor genes within the gastric mucosa. Consequently, it impedes the natural healing process of ulcers [111]. TNF-α, a cytokine, is responsible for facilitating the transcription of various adhesion molecules. This process ultimately results in the observed infiltration of inflammatory cells in rats treated with indomethacin. Moreover, the infiltrating leukocytes play a significant role in the production of ROS, which can further disrupt the oxidative balance. IL-1β, a cytokine with pro-inflammatory properties, plays a crucial role in the regulation of multiple genes associated with the inflammatory response and the consequent tissue damage. This includes the disruption of enterochromaffin-like cell function. Furthermore, it has been observed that IL-1β exerts an inhibitory effect on the proliferation of gastric epithelial cells [112]. Remarkably, the extract derived from *A. graveolens* exhibited a notable improvement in the infiltration of inflammatory cells, while also significantly reducing the heightened levels of TNF-α, IL-1β, and IL-6 in the gastric contents of rats treated with indomethacin.

In previous investigations, it was observed that the production of TNF-α has the potential to enhance the production of nitric oxide (NO) through the overexpression of iNOS in indomethacin-induced jejunoileitis [113]. NO which is generated by iNOS, assumes a significant role in the process of ulcer formation. This is primarily achieved through the generation of peroxynitrite (ONOO-) and subsequent cellular toxicity, protein tyrosine nitration, production of hydroxyl radicals (HO.), and consequent tissue damage [114]. The current investigation demonstrated that the administration of indomethacin resulted in an upregulation of iNOS expression within the gastric tissues. This observation potentially correlates with the upregulation of TNF-α synthesis within the gastric tissues. The extract exhibited a level of inhibition in the expression of iNOS that was comparable to that of famotidine. This inhibition effectively prevented the excessive release of NO, which is known to worsen gastric mucosal injury. As a result, the extract contributed to the improvement of ulcer healing.

Pharmacology networking study was carried out to investigate the chemical-biological interactions between the identified metabolites of *A. graveolens* L. seed extract and gastric ulcer. The pharmacology network explained target genes related to the identified metabolites to possess 672 interactions between the 18 identified metabolites and 379 genes, among which PTGS2, MMP2 and PTGS1 were the top annotated genes related to gastric ulcer. The pharmacology network determined the top KEGG pathways of *A. graveolens* L. The PPI network identified VEGFA, HIF1A, TP53, and EGFR as the genes with high interactions. The top KEGG biological pathway according to fold enrichment was the bladder cancer and the top signaling pathway was the VEGF signaling pathway. These results assure the ability of the *Apium graveolens* L. to influence the gastric ulcer by 17 genes and determined the biological pathways.

Taken together, this study is the first to highlight the role of *Apium graveolens* L. in indomethacin-induced gastric ulcer, and correlate it to IKκB/NF-κB p65/IL1β, IL-6, TNF-α/iNOS signaling pathways. Additionally, it presents scientific evidence regarding the potential gastroprotective activity exhibited by *Apium graveolens* L. Furthermore, it reinforces the significance of *Apium graveolens* L. as a promising natural supplement with anti-ulcerative properties. Therefore, the clinical significance of investigating natural products combating gastric ulcers resides in their potential to offer efficacious, secure, and readily available therapeutic alternatives for the prevention and management of gastric ulcers.

## Conclusions

The present study has demonstrated the noteworthy gastroprotective effects of the seed extract derived from *A. graveolens*. These effects were observed in the context of indomethacin-induced gastric mucosal injury, and were found to be comparable to the effects exhibited by famotidine, which was used as the reference drug. Additionally, it showcased the presence of antioxidant properties and robust anti-inflammatory effects, which are likely facilitated through the inhibition of IKκB/NF-κB p65/IL1β, IL-6, TNF-α/iNOS signaling pathways. This inhibition aids in the prevention of reducing gastric acidity, thereby overcoming the adverse effects associated with chemical anti-secretory medications commonly employed in clinical settings.

### Supplementary Information

**Additional file 1. Figure S1.** LC-ESI-HR-MS total ion chromatogram of *A. graveolens* L. seed extract, taken in positive ionization mode. **Figure S2.** LC-ESI-HR-MS total ion chromatogram of *A. graveolens* L. seed extract, taken in negative ionization mode. **Table S1.** List of tentatively identified metabolites and dereplicated from LC–HR– ESI–MS of *Apium graveolens* L. seeds. **Table S2.** Top gene enrichment analysis of genes annotated by *Apium graveolens* L. metabolites and related to gastric ulcer in terms of biological process, cellular component and molecular function. **Table S3.** Top 20 KEGG biological pathways of genes annotated by *Apium graveolens* L. metabolites and related to gastric ulcer.

## Data Availability

All data generated or analyzed during this study are included in this article (and its supplementary information files).
