# Supplementary material for: Apium extract alleviates indomethacin-induced gastric ulcers in rats via modulating the VEGF and IK-κB/NF-κB p65 signaling pathway: insights from in silico and in vivo investigations
Source: BMC Complement Med Ther. 2024 Feb 14;24:88. doi: 10.1186/s12906-023-04333-w (PMC10865661; doi:10.1186/s12906-023-04333-w)
Supplement: Supplementary file 1 — Additional file 1. Figure S1. LC-ESI-HR-MS total ion chromatogram of A. graveolens L. seed extract, taken in positive ionization mode. Figure S2. LC-ESI-HR-MS total ion chromatogram of A. graveolens L. seed extract, taken in negative ionization mode. Table S1. List of tentatively identified metabolites and dereplicated from LC–HR– ESI–MS of Apium graveolens L. seeds. Table S2. Top gene enrichment analysis of genes annotated by Apium graveolens L. metabolites and related to gastric ulcer in terms of biological process, cellular component and molecular function. Table S3. Top 20 KEGG biological pathways of genes annotated by Apium graveolens L. metabolites and related to gastric ulcer. [file 12906_2023_4333_MOESM1_ESM.docx]

**Supplementary Information**

Apium Extract alleviates Indomethacin-Induced Gastric Ulcers in Rats via Modulating the VEGF and IK-κB/NF-κB p65 Signaling Pathway: Insights from In Silico and In Vivo Investigations

Dalia H. Abu-Baih^a,†^, Alshymaa Abdel-Rahman Gomaa^b,†^, Nada Mohamed Abdel-Wahab^b,†^, Enas Reda Ab-delaleem^b,†^, Azza M. Abdel Zaher^c^, Noha F. Hassan^d^, Gerhard Bringman^e^, Usama Ramadan Abdelmohsen^b, f, *^, Faisal H. Altemani^g^, Naseh A. Algehainy^g^, Fatma Alzahraa Mokhtar^h^, Miada F. Abdelwahab^b^

^a^Department of Biochemistry, Faculty of Pharmacy, Deraya University, New Minia 61111, Egypt

^b^Department of Pharmacognosy, Faculty of Pharmacy, Minia University, Minia 61519, Egypt

^c^Department of Pathology, Faculty of Medicine, Minia University, Minia, Egypt

^d^Department of Pharmacology and Toxicology, Faculty of Pharmacy, Modern University for Technology and Information, Cairo11571, Egypt,

^e^Institute of Organic Chemistry, University of Würzburg, Am Hubland, 97074 Würzburg, Germany

^f^Department of Pharmacognosy, Faculty of Pharmacy, Deraya University, Minia 61111, Egypt

^g^Department of Medical Laboratory Technology, Faculty of Applied Medical Sciences, University of Tabuk, Tabuk 71491, Saudi Arabia.

^h^Department of Pharmacognosy, Faculty of Pharmacy, Al Salam University, Kafr Alzayat 31611, Al Gharbia, Egypt.

*Correspondence: usama.ramadan@mu.edu.eg (U.R.A.)

†These authors contributed equally to this work

Abstract: Gastric ulcers represent a worldwide health problem, characterized by erosions that affect the mucous membrane of the stomach and may even reach the muscular layer, leading to serious complications. Numerous natural products have been assessed as anti-ulcerogenic and/or gastroprotective agents, and have been considered as new approaches to the complementary treatment and/or prevention of gastric ulcers. In the current study, we have explored the protective effects of the seed extract of Apium graveolens L. (Apiaceae), commonly known as celery, against indomethacin-induced gastric ulcers in rats. Metabolomic profiling, employing liquid chromatography coupled to high-resolution electrospray ionization mass spectrometry (LC-HR-ESI-MS), was implemented with the aim of investigating the chemical profile of the seeds. The in vivo evaluation was further confirmed by histopathological examination of the gastric tissues as well as assessment of various inflammatory cytokines, nitrosative stress and oxidative stress markers. Pre-treatment with A. graveolens seed extract improved gastric mucosal damage, as evidenced by the significant reduction in the ulcer index (0.28) compared to the indomethacin group (p < 0.05), with effects similar to those reported from the reference drug famotidine (0.3). Additionally, the gastric acidity was reduced with no significant difference between the famotidine and A. graveolens extract treated groups (p > 0.05). Moreover, the gastroprotective effect was demonstrated through examination of the level of malondialdehyde (MDA), which was significantly reduced (p < 0.05), as well as the level of gastric reduced glutathione (GSH) which was significantly increased (p < 0.05) upon pre-treatment with A. graveolens seed extract. Vascular endothelial growth factor (VEGF), a fundamental angiogenic factor that stimulates angiogenesis, was markedly inhibited by indomethacin. A. graveolens seed extract restored this diminished level of VEGF. The IKκB/NF-κB p65 signaling cascade, triggered by indomethacin, was markedly attenuated, as manifested by the remarkable decrements in IKκB and NF-κB protein levels compared to the injured mucosa. These activities were also correlated to the tentatively featured secondary metabolites including, phenolic acids, coumarins and flavonoids, previously evidenced to exert potent anti-inflammatory and antioxidant activities. According to our network pharmacology study, the identified metabolites annotated 379 unique genes, among which only 17 genes were related to gastric ulcer. The PTGS2, MMP2 and PTGS1 were the top annotated genes related to gastric ulcer. The top biological pathway was the VEGF signaling pathway. In conclusion, A. graveolens seed extract possesses significant anti-ulcer activity, similar to famotidine, against indomethacin-induced gastric lesions in rats. It is worth highlighting that the extract does not reduce gastric acidity, and thus it overcomes the side effects of the conventional chemical anti-secretory drugs.

Keywords: Apium graveolens L.; gastric ulcer; metabolomic profiling; network pharmacology; inflammatory cytokines.


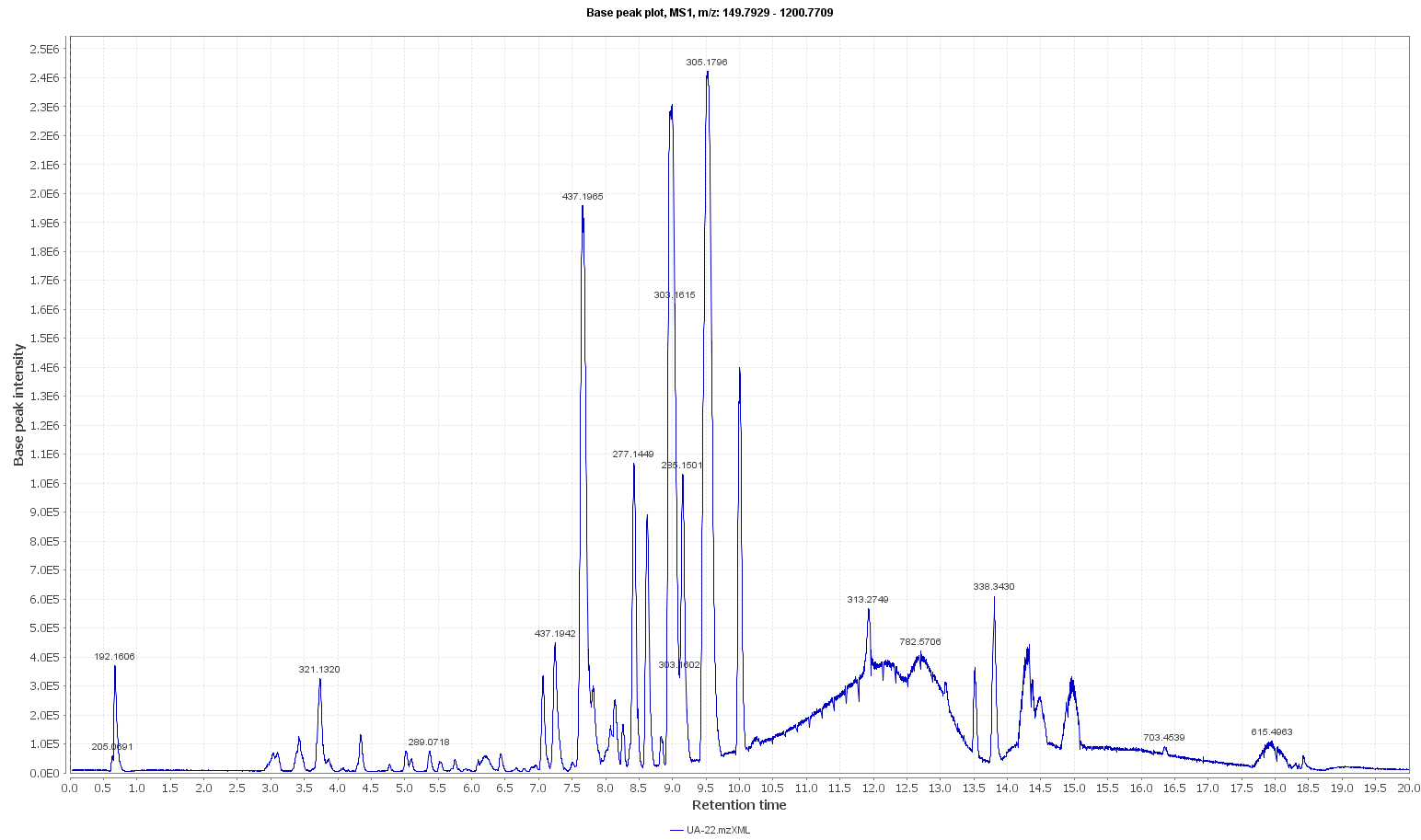


**Figure S1.** LC-ESI-HR-MS total ion chromatogram of *A. graveolens* L. seed extract, taken in positive ionization mode.


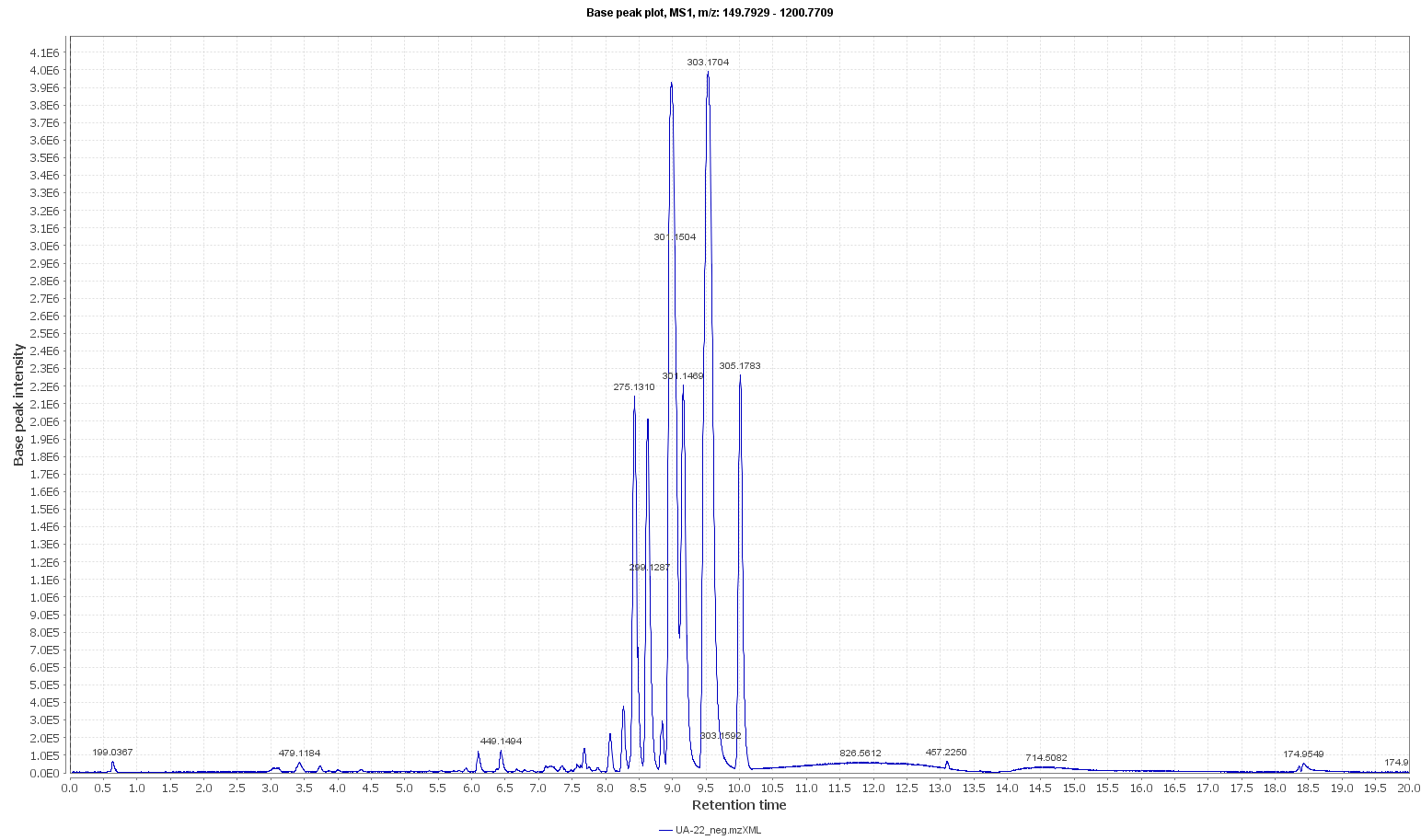


**Figure S2.** LC-ESI-HR-MS total ion chromatogram of *A. graveolens* L. seed extract, taken in negative ionization mode.

**Table S1.** List of tentatively identified metabolites and dereplicated from LC–HR– ESI–MS of *Apium graveolens* L. seeds.

| **No.** | **Compound** | **Retention**  **time (min)** | ***m/z*** | **Ionization mode** | **Accurate mass** | **Molecular formula** | **References** |
| --- | --- | --- | --- | --- | --- | --- | --- |
| **Phenolic acids** | |  |  |  |  |  |  |
| **1** | 3-Methoxy-4,5-methylenedioxybenzoic acid  (Myristicic acid) | 2.28 | 197.044 | Positive | 196.0376 | C_9_H_8_O_5_ | [56] |
| **2** | Chlorogenic acid | 13.43 | 353.305 | Negative | 354.3125 | C_16_H_18_O_9_ | [57] |
| **3** | 2,3-Dihydro-6-hydroxy-2-methyl-5-benzofurancarboxylic  acid | 7.17 | 195.066 | Positive | 194.0587 | C_10_H_10_O_4_ | [58] |
| **Coumarins** | |  |  |  |  |  |  |
| **4** | Celerin | 7.47 | 261.112 | Positive | 260.1055 | C_15_H_16_O_4_ | [59] |
| **5** | Osthenol | 9.13 | 231.101 | Positive | 230.0946 | C_14_H_14_O_3_ | [32, 60] |
| **6** | 6-Acetyl-7-hydroxy-2*H*-1-benzopyran-2-one | 6.16 | 203.033 | Negative | 204.040 | C_11_H_8_O_4_ | [61] |
| **7** | 7-(2-Hydroxy-3-methyl-3-butenyloxy)-6-methoxycoumarin  (Virgatenol) | 4.58 | 275.091 | Negative | 276.0985 | C_15_H_16_O_5_ | [62] |
| **8** | 10-*O*-Tigloylkhellactone | 8.39 | 343.114 | Negative | 344.1222 | C_19_H_20_O_6_ | [63] |
| **Furanocoumarins** | |  |  |  |  |  |  |
| **9** | 2,3-dihydro-2(1-hydroxy-1-hydroxy-methylethyl)-7H-furo[3,2g][1]benzopyran-7-one  (Dorsteniol) | 9.48 | 263.128 | Positive | 262.1212 | C_14_H_14_O_5_ | [32, 64] |
| **10** | 5-Methoxy-8-*O*-*β*-D-glucosyloxypsoralen | 14.40 | 395.331 | Positive | 394.3240 | C_18_H_18_O_10_ | [65] |
| **11** | Oxypeucedanin | 7.37 | 287.092 | Positive | 286.0847 | C_16_H_14_O_5_ | [66] |

| **Phthalides** | |  |  |  |  |  |  |
| --- | --- | --- | --- | --- | --- | --- | --- |
| **12** | 3'-*β*-D-Glucopyranosyloxy, 7,7*a*-didehydrobutylhexahydro-1(3*H*) isobenzofuranone  (Celephthalide C) | 8.28 | 373.194 | Positive | 372.1869 | C_18_H_28_O_8_ | [67] |
| **13** | 3-(3-Methylbutylidene)-1(3*H*)-isobenzofuranone  (3-Isovalidenephthalide) | 9.37 | 203.106 | Positive | 202.0997 | C_13_H_14_O_2_ | [68] |
| **14** | 3a,4-Dihydro-3-(3-methylbutylidene)-  1(3H) isobenzofuranone  (3-Isovalidene-3a,4-dihydrophthalide) | 9.39 | 205.123 | Positive | 204.1157 | C_13_H_16_O_2_ | [32, 68] |
| **Sesquiterpenes** | |  |  |  |  |  |  |
| **15** | 4(15)-Eudesmene-1,2,11-  triol 11-*O*-glucoside  (Celerioside D) | 9.53 | 417.240 | positive | 416.2329 | C_21_H_36_O_8_ | [32, 67] |
| **Polyacetylenes** | |  |  |  |  |  |  |
| **16** | 1,9-Heptadecadiene-4,6-diyne-3,8-diol  (Falcarindiol) | 9.57 | 261.185 | Positive | 260.1778 | C_17_H_24_O_2_ | [69] |
| **Flavonoids** | |  |  |  |  |  |  |
| **17** | Luteolin | 11.85 | 287.237 | Positive | 286.2304 | C_15_H_10_O_6_ | [70] |
| **Fatty acids** | |  |  |  |  |  |  |
| **18** | 6-Octadecenoic acid | 12.72 | 281.247 | Negative | 282.2548 | C_18_H_34_O_2_ | [71] |

**Table S2.** Top gene enrichment analysis of genes annotated by *Apium graveolens* L. metabolites and related to gastric ulcer in terms of biological process, cellular component and molecular function.

| **No**. | **Pathway** | **Fold** **enrichment** | **nGenes** | **Genes** |
| --- | --- | --- | --- | --- |
| **Biological process** | | | | |
| 1 | Pos. reg. of endothelial cell chemotaxis | 268.188 | 3 | MET VEGFA KDR |
| 2 | Cyclooxygenase pathway | 243.807 | 2 | PTGS2 PTGS1 |
| 3 | Cellular response to UV-A | 243.807 | 2 | MMP2 MMP9 |
| 4 | Response to UV-A | 191.563 | 2 | MMP2 MMP9 |
| 5 | Pos. reg. of cell migration involved in sprouting angiogenesis | 182.856 | 3 | PTGS2 VEGFA KDR |
| 6 | Embryonic hemopoiesis | 174.905 | 3 | HIF1A VEGFA KDR |
| 7 | Endothelial cell chemotaxis | 138.718 | 3 | MET VEGFA KDR |
| 8 | Peptidyl-tyrosine autophosphorylation | 138.718 | 3 | VEGFA KDR ACE |
| 9 | Mammary gland alveolus development | 134.094 | 2 | HIF1A VEGFA |
| 10 | Pos. reg. of blood vessel endothelial cell migration | 124.161 | 5 | PTGS2 HIF1A VEGFA KDR ADAM17 |
| 11 | Vascular endothelial cell proliferation | 121.904 | 2 | PPARG ADAM17 |
| 12 | Reg. of vascular endothelial cell proliferation | 121.904 | 2 | PPARG ADAM17 |
| 13 | Serotonin secretion | 121.904 | 1 | HRH3 |
| 14 | Hydrogen peroxide mediated programmed cell death | 121.904 | 1 | MET |
| 15 | Gas homeostasis | 121.904 | 1 | HIF1A |
| 16 | Hormone catabolic proc. | 121.904 | 1 | ACE |
| 17 | Hemoglobin biosynthetic proc. | 121.904 | 1 | HIF1A |
| 18 | Bone trabecula formation | 121.904 | 1 | MMP2 |
| 19 | Macrophage proliferation | 121.904 | 1 | MAPK1 |
| 20 | Reg. of chemokine mediated signaling pathway | 121.904 | 1 | HIF1A |
| **Cellular component** | | | | |
| 1 | Integral component of membrane | 273.161 | 1 | MET |
| 2 | Intrinsic component of membrane | 273.161 | 1 | MET |
| 3 | Membrane | 136.580 | 1 | MET |
| 4 | Cellular anatomical entity | 57.507 | 1 | MET |
| **Molecular function** | | | | |
| 1 | Nucleotide binding | 1 | 364.216 | MET |
| 2 | ATP binding | 1 | 364.216 | MET |
| 3 | Transferase activity | 1 | 364.216 | MET |
| 4 | Purine nucleotide binding | 1 | 364.216 | MET |
| 5 | Adenyl nucleotide binding | 1 | 364.216 | MET |
| 6 | Ribonucleotide binding | 1 | 364.216 | MET |
| 7 | Purine ribonucleotide binding | 1 | 364.216 | MET |
| 8 | Adenyl ribonucleotide binding | 1 | 364.216 | MET |
| 9 | Purine ribonucleoside triphosphate binding | 1 | 364.216 | MET |
| 10 | Small molecule binding | 1 | 364.216 | MET |
| 11 | Anion binding | 1 | 364.216 | MET |
| 12 | Carbohydrate derivative binding | 1 | 364.216 | MET |
| 13 | Nucleoside phosphate binding | 1 | 364.216 | MET |
| 14 | Ion binding | 1 | 218.529 | MET |
| 15 | Organic cyclic compound binding | 1 | 218.529 | MET |
| 16 | Heterocyclic compound binding | 1 | 218.529 | MET |
| 17 | Catalytic activity, acting on a protein | 1 | 182.108 | MET |
| 18 | Catalytic activity | 1 | 121.405 | MET |
| 19 | Binding | 1 | 109.265 | MET |

**Table S3.** Top 20 KEGG biological pathways of genes annotated by *Apium graveolens* L. metabolites and related to gastric ulcer.

| **Pathway** | **Fold** **Enrichment** | **nGenes** | **Genes** |
| --- | --- | --- | --- |
| Bladder cancer | 130.824 | 4 | MMP2 MAPK1 MMP9 VEGFA |
| VEGF signaling pathway | 90.911 | 4 | PTGS2 MAPK1 VEGFA KDR |
| Renal cell carcinoma | 78.879 | 4 | MAPK1 HIF1A MET VEGFA |
| Thyroid cancer | 72.483 | 2 | MAPK1 PPARG |
| EGFR tyrosine kinase inhibitor resistance | 67.896 | 4 | MAPK1 MET VEGFA KDR |
| Arachidonic acid metabolism | 65.948 | 3 | PTGS2 PTGS1 CYP2C19 |
| Serotonergic synapse | 59.863 | 5 | PTGS2 PTGS1 MAPK1 CASP3 CYP2C19 |
| Renin-angiotensin system | 58.302 | 1 | ACE |
| IL-17 signaling pathway | 57.675 | 4 | PTGS2 MAPK1 MMP9 CASP3 |
| Epithelial cell signaling in *Helicobacter pylori* infection | 57.469 | 3 | MET ADAM17 CASP3 |
| Central carbon metabolism in cancer | 57.469 | 3 | MAPK1 HIF1A MET |
| Platinum drug resistance | 55.107 | 3 | MAPK1 GSTM1 CASP3 |
| AGE-RAGE signaling pathway in diabetic complications | 53.638 | 4 | MMP2 MAPK1 VEGFA CASP3 |
| Proteoglycans in cancer | 53.107 | 8 | MMP2 MAPK1 HIF1A MMP9 MET VEGFA KDR CASP3 |
| MicroRNAs in cancer | 49.973 | 6 | PTGS2 MAPK1 MMP9 MET VEGFA CASP3 |
| Fluid shear stress and atherosclerosis | 48.585 | 5 | MMP2 MMP9 VEGFA KDR GSTM1 |
| TNF signaling pathway | 47.891 | 4 | PTGS2 MAPK1 MMP9 CASP3 |
| Reg. of lipolysis in adipocytes | 47.891 | 2 | PTGS2 PTGS1 |
| Linoleic acid metabolism | 46.239 | 1 | CYP2C19 |
| Endocrine resistance | 42.346 | 3 | MMP2 MAPK1 MMP9 |
